# Metabolically engineered plant cell cultures as biofactories for the production of high-value carotenoids astaxanthin and canthaxanthin

**DOI:** 10.1038/s41598-025-11916-9

**Published:** 2025-08-06

**Authors:** Bárbara A. Rebelo, M. Rita Ventura, Rita Abranches

**Affiliations:** 1 Plant Cell Biology Laboratory, Instituto de Tecnologia Química e Biológica António Xavier (ITQB NOVA), Universidade Nova de Lisboa, Oeiras, 2780-157 Portugal; 2 Bioorganic Chemistry Laboratory, Instituto de Tecnologia Química e Biológica António Xavier (ITQB NOVA), Universidade Nova de Lisboa, Oeiras, 2780-157 Portugal

**Keywords:** Molecular farming, *Nicotiana tabacum* BY-2 non-photosynthetic cells, Metabolic engineering, Synthetic biology, Sustainable bioproduction, Secondary metabolites, Secondary metabolism, Biosynthesis, Molecular engineering in plants, Metabolic engineering, Expression systems

## Abstract

Astaxanthin and canthaxanthin are high-value carotenoids with growing demand due to their antioxidant properties and applications in food, cosmetic, and pharmaceutical sectors. However, natural sources are limited and current production methods are often costly or unsustainable. In this study, we developed a plant-based platform for ketocarotenoid biosynthesis using metabolically engineered *Nicotiana tabacum* BY-2 cell suspension cultures. Specifically, we expressed a marine bacterial *crtW* gene (*β-carotene ketolase*) alone or in combination with overexpressed plant *psy* (*phytoene synthase*) and *crtI* (*phytoene desaturase*) genes. The resulting cell lines displayed visually distinct pigmentation and accumulated different ketocarotenoid profiles based on their genetic modifications. Single-gene transformants expressing *crtW* produced up to 50 µg g⁻¹ DW of canthaxanthin and 127 µg g⁻¹ DW of astaxanthin. Co-expression of all three genes significantly increased canthaxanthin accumulation to 788 µg g⁻¹ DW. Our results establish suspended undifferentiated plant cells as a scalable and sustainable system for ketocarotenoid production, offering a biological alternative to natural producers and chemical synthesis.

**Supplementary Information:**

The online version contains supplementary material available at 10.1038/s41598-025-11916-9.

## Introduction

Plant-derived secondary metabolites, such as carotenoids, have significant applications for humans and animals. Among them, carotenoids are of particular importance for living systems due to their antioxidant properties and protective action against photooxidative damage^1,2^. These natural pigments have attracted considerable interest for nutraceuticals and functional foods, as consumers seek natural sources of these valuable antioxidants for improved health and wellness^3^. Carotenoids are also widely used in cosmetics and skincare industries that take advantage of their anti-aging and UV-protective properties, meeting growing preferences for natural and sustainable ingredients^4^. Additionally, pharmaceutical companies incorporate carotenoids into formulations for the prevention or treatment of various chronic diseases, including cardiovascular diseases, diabetes, and eye diseases^4–6^. Despite their diverse benefits and growing market relevance, natural carotenoids remain limited by supply constraints and high production costs, motivating the search for alternative, scalable, and sustainable production systems. Among the various carotenoids, high-value pink-to-red ketocarotenoids such as canthaxanthin and astaxanthin represent a subgroup of carotenoids widely used in nutraceutical, cosmetic and animal feed industries^7,8^. These compounds are structurally distinguished by the presence of one or more ketones, which are functional groups containing a carbonyl moiety. The characteristic color of ketocarotenoids is determined by their structure, in which the conjugated double-bond structure acts as a light-absorbing chromophore and the two keto groups shift the absorption maximum to higher wavelengths^8^.

**Fig. 1:**
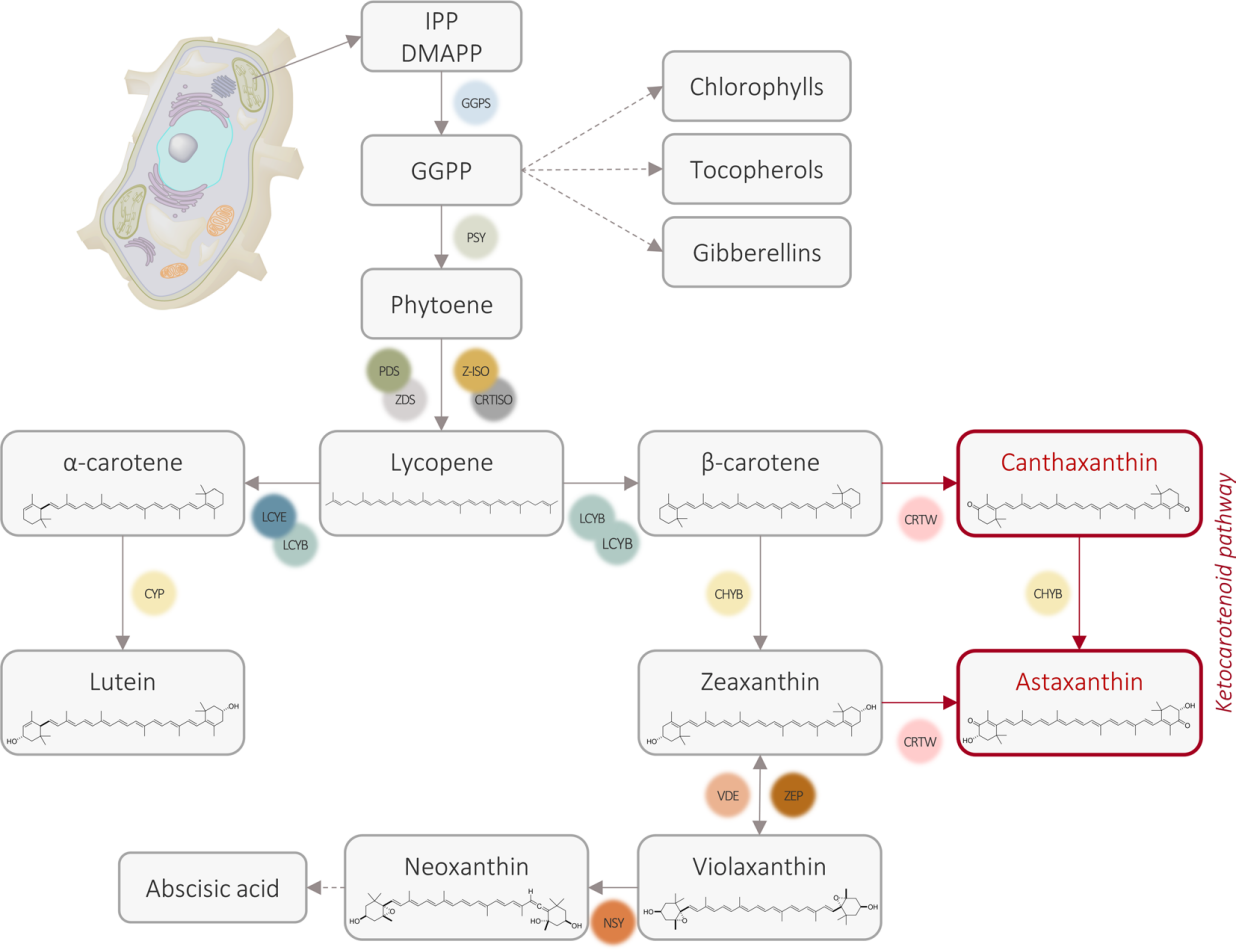
Schematic overview of the carotenoid biosynthetic pathway in plants. The isopentenyl diphosphate (IPP) and dimethylallyl diphosphate (DMAPP) building blocks can be produced from the methylerythritol phosphate (MEP) cytoplasmic and mevalonate (MVA) plastid pathways. The grey boxes indicate the main carotenoids and derivatives found in plant systems, while the red boxes indicate the heterologous ketocarotenoid pathway. Abbreviations of enzymes: GGPS (geranylgeranyl pyrophosphate synthase), PSY (phytoene synthase, CRTB for bacteria), PDS (phytoene desaturase, CRTI for bacteria), ZDS (ζ-carotene desaturase, CRTI for bacteria), Z-ISO (ζ-carotene isomerase, CRTI for bacteria), CRTISO (carotenoid isomerase, CRTI for bacteria), LCYE (lycopene ε-cyclase), LCYB (lycopene β-cyclase, CRTY for bacteria), CYP (cytochrome P450 enzyme), CHYB (β-carotene hydroxylase, BHY for algae, CRTZ for bacteria), CRTW (β-carotene ketolase, BKT for algae), VDE (violaxanthin de-epoxidase), ZEP (zeaxanthin epoxidase), NSY (neoxanthin synthase). This visual representation shows the stepwise process involved in ketocarotenoid synthesis.

Regarding the chemical structure, canthaxanthin (β,β-carotene-4,4’-dione) results from the modification of β-carotene by the β-carotene ketolase enzyme, which adds two oxo substituents at 4- and 4’-positions of the β-ionone backbone. Canthaxanthin is an important pigment in itself, but it is also the substrate for obtaining astaxanthin. The conversion is catalyzed by the enzyme β-carotene hydroxylase, which introduces two hydroxyl moieties at the 3- and 3’-positions of canthaxanthin cyclic rings, affording astaxanthin. Alternatively, β-carotene can be first hydroxylated by the same enzyme (β-carotene hydroxylase) to form zeaxanthin. This intermediate can then be converted into astaxanthin by the action of the β-carotene ketolase enzyme (Fig. 1), which adds two oxo substituents at 4- and 4’-positions of the β-ionone backbone^8–10^. The resulting astaxanthin contains both hydroxy and keto functional groups, enhancing its antioxidant capacity compared with zeaxanthin^8,10^.

Interestingly, the ketolation reaction necessary to produce these ketocarotenoids is only performed in nature by a few species of algae and microorganisms^8^. Examples of natural producers include the microalgae *Haematococcus pluvialis* and *Chromochloris zofingiensis*, the bacteria *Brevundimonas* sp. SD212, and the yeast *Phaffia rhodozyma*. The only known plant genus which naturally produces ketocarotenoids is *Adonis*, that is able to accumulate high levels of astaxanthin in its flower petals^11^. However, in contrast to the typical pathway involving β-carotene hydroxylase and ketolase, this plant species utilizes very different enzymes, the β-ring 4-dehydrogenase (CBFD) and 4-hydroxy-β-ring 4-dehydrogenase (HBFD).

Natural sources of ketocarotenoids are associated with high production costs, insufficient quantities, and complex isolation procedures^10^. For these reasons, the feed industry often resorts to chemical synthesis for supply^8,12^raising sustainability concerns due to its reliance on petrochemical-derived precursors. Because of the structural complexity of ketocarotenoids, their production by chemical synthesis requires long synthetic routes and highly stereoselective reactions, making large-scale production challenging and costly^13–15^.

Since biological production of ketocarotenoids cannot economically compete with synthetic production, and the latter is not an option for human applications, various strategies have been employed to improve ketocarotenoid production in biological systems. Several strategies of genetic modification have been accomplished, leading to carotenoids with variable qualitative and quantitative profiles (reviewed in^1,8,9^). A noteworthy example is the orange-red-grained astaxanthin rice^16^obtained through transformation with multiple carotenogenic genes: plant *phytoene synthase* (*psy*), bacterial *phytoene desaturase* (*crtI*) and algal *β-carotene hydroxylase* (*bhy*) and *ketolase* (*bkt*). These plants are capable of producing astaxanthin up to 16 µg g^−1^ DW^16^. In another study, *Nicotiana glauca* was transformed with bacterial *β-carotene hydroxylase* and a *ketolase* gene (*crtZ* and *crtW*) from *Brevundimonas* sp., resulting in astaxanthin production of 140 µg g^−1^ DW^17^. In another report, using the same enzymes in tomato, an even higher astaxanthin yield of 362 µg g^−1^ DW^18^ was achieved. The same authors improved the ketocarotenoid content in tomato by crossing a low ketocarotenoid producing line (ZW)^18^ with an orange fruit recombinant inbred line that accumulated high levels of β-carotene^15,19^. More recently, ketocarotenoid production in chloroplast-engineered tobacco plants expressing a *bkt* gene in combination with the *lycopene cyclase* (*lcy*) and *bhy* genes was achieved. However, higher xanthophyll levels were obtained, rather than ketocarotenoids, and only 4-ketolutein was reported^20^. Using a different approach, *Nicotiana benthamiana* plants were engineered with β-ring 4-dehydrogenase (CBFD) and 4-hydroxy-β-ring 4-dehydrogenase (HBFD) enzymes from *Adonis aestivalis*, in order to produce astaxanthin directly from β-carotene^21^.

Microbial systems have also been used to produce ketocarotenoids, including bacteria, algae, and yeasts. There are two industrially important natural sources, *P. rhodozyma* and *H. pluvialis*. *P. rhodozyma* can have lower cultivation costs compared to certain plant-based systems but it accumulates astaxanthin in cytoplasmatic droplets associated with the membrane and fatty acids, and has a thick yeast cell wall, making extraction inefficient and costly^22,23^. *H. pluvialis* is the preference for large scale astaxanthin production with its extracts approved as food ingredients. However, its life cycle is quite complex and the induction of astaxanthin accumulation (red non-motile cell with tick cell wall) requires high light intensity, controlled nutrient depletion, and prolonged growth periods. This limits productivity and contributes to a higher price for natural astaxanthin^23–25^.

The yeast *Saccharomyces cerevisiae*, a recognized GRAS organism, has been engineered for astaxanthin production. However, the complexity of the carotenoid pathway presents a significant challenge as it requires the coordinated expression of multiple heterologous genes encoding for enzymes that catalyze the conversion of geranylgeranyl pyrophosphate (GGPP) through a series of intermediates to astaxanthin. Moreover, it may require precursor supply optimization, enhancing enzyme efficiency and minimizing the accumulation of by-products^26^. Being a non-oleaginous organism, *S. cerevisiae* contains few and small lipid droplets, leading to limited storage capacity for lipophilic compounds such as astaxanthin^26,27^. Other systems have also shown promising results. For example, *Chlamydomonas reinhardtii* has been modified by overexpressing either endogenous or *P. rhodozyma β-carotene ketolase* genes, resulting in elevated levels of β-carotene and astaxanthin by 1.8-fold and 1.2-fold, respectively^28^. In another study, combining *C. reinhardtii* β-carotene *bkt* with *phytoene synthase* (*crtB*) from *Pantoea ananatis*, and *C. reinhardtii β-carotene 3-hydroxylase* (*chyb*)^29^ resulted in a volumetric astaxanthin production of 9.5 mg L^−1^ (4.5 mg g^−1^ cell dry weight (DW)) under mixotrophic conditions and 23.5 mg L^−1^ (1.09 mg L^−1^ h^−1^) under high cell density conditions.

Overall, recent advancements in plant synthetic biology provide abundant genetic materials and engineering strategies to create opportunities for tailored cell factories for specific applications^30^. In this context, plant-based systems for carotenoid production offer environmentally friendly alternatives to traditional chemical synthesis methods, aligning with the increasing consumer preference for natural and sustainable products. In the present study, we used metabolic engineering to produce ketocarotenoids in plant cell suspension cultures, namely tobacco BY-2 cells. Carotenoid production, without substrate feeding, was achieved by expressing various combinations of carotenoid-related genes. This combinatorial transformation resulted in colored BY-2 cells that exhibited continuous pigment production throughout subculturing, while maintaining normal cell growth and developmental process. By using this platform, it is possible to produce ketocarotenoids in a reliable, reproducible, efficient way, with rentable yields, whilst maintaining an environmentally friendly sustainable process that addresses the limitations of other systems.

## Results

### Vector construction for the expression of selected genes involved in carotenoid biosynthesis

**Fig. 2:**
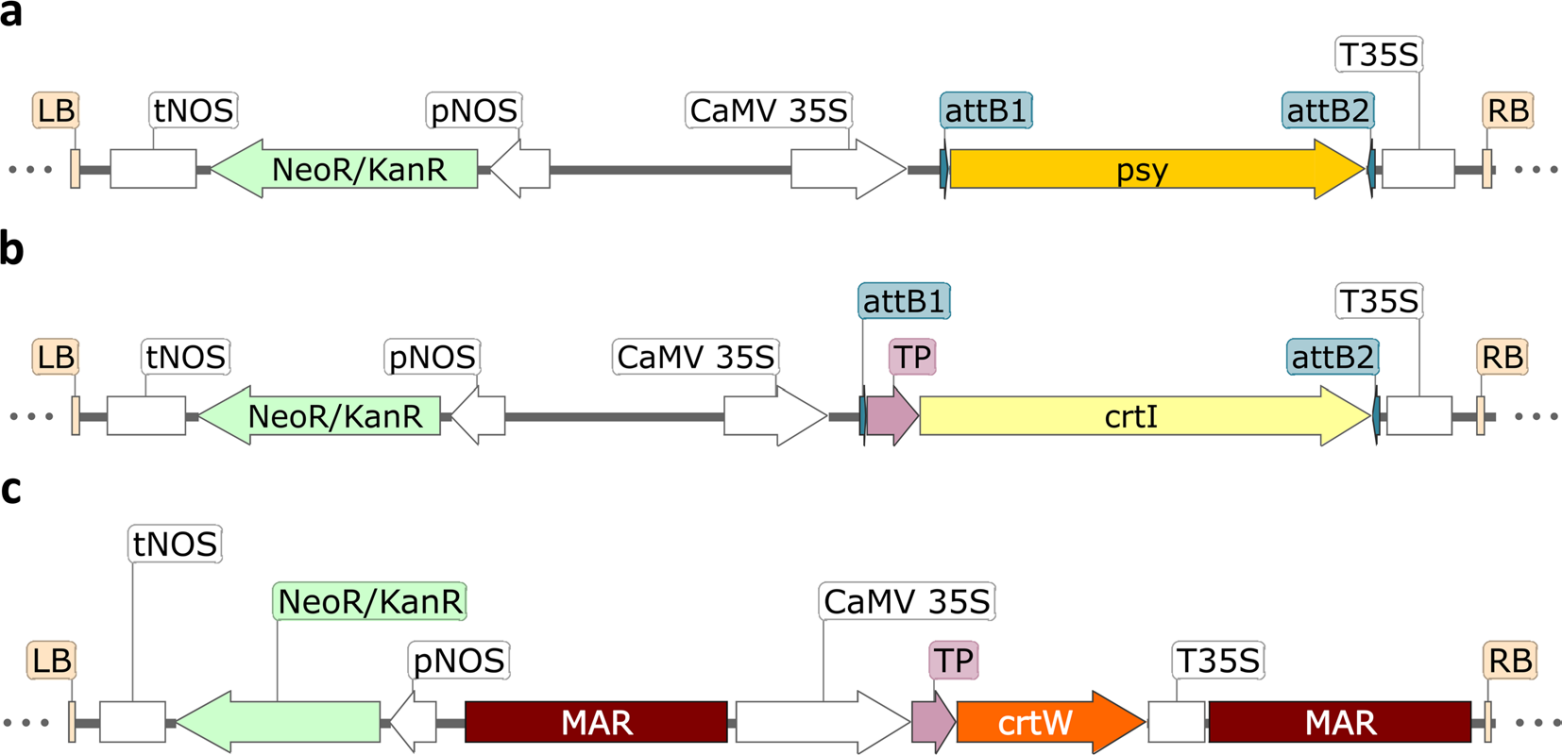
Schematic representation of the T-DNA cassette of **(a)** construct pY, **(b)** construct pI and **(c)** construct pW used for the transformation of BY-2 cells. Regions are represented as follows: left border (LB), nopaline synthase terminator (tNOS), kanamycin resistance marker (NeoR/KanR), nopaline synthase promoter (pNOS), matrix attachment regions (MAR), Cauliflower Mosaic Virus 35 S promoter (CaMV 35 S), site-specific recombination site (attB1 and attB2), transit peptide (TP), *phytoene synthase* coding sequence (AY324431), *phytoene desaturase* coding sequence (D90087), *β-carotene ketolase* coding sequence, 35 S terminator (T35S), right border (RB). All dark lines are nucleotide sequences without relevant features.

The non-green *Nicotiana tabacum* BY-2 cell line, although widely used in fundamental research, has not been previously explored for ketocarotenoid biosynthesis. There are only a few reports on the heterologous biosynthesis of pigments in these cultured cells^31–33^. In this work, we expressed key carotenogenic genes to complete and enhance the carotenoid pathway for the production of astaxanthin and canthaxanthin. We selected the *β-carotene ketolase* gene (*crtW*) from the marine bacteria *Brevundimonas* sp. strain SD212, known for efficiently catalyzing ketocarotenoid biosynthesis. Since BY-2 cells lack an endogenous *β-carotene ketolase* gene in their genome, the introduction of this gene was essential. To increase precursor supply, such as phytoene and lycopene, we co-expressed the *phytoene desaturase* gene (*crtI*) from *Pantoea ananatis* and the *phytoene synthase* gene (*psy*) from *Zea mays*. Previous studies have shown that boosting substrate production upstream of the ketocarotenoid pathway can enhance carotenoid biosynthesis yields. The CRTI was preferred over plant desaturases enzymes because it catalyzes multiple desaturation steps (Fig. 1) within a single enzyme, simplifying pathway engineering. The *psy* or *crtI* genes were inserted into the binary vector pK2GW7, while the synthetic *crtW* gene was cloned into the pTRA vector. This resulted in constructs pY, pI and pW (Fig. 2), with all genes under the control of the 35 S promoter from cauliflower mosaic virus to ensure robust expression. The constructs were named based on the last letter of each gene included in the cassette. To direct the enzyme to the plastids, a plastid-transit peptide from the pea Rubisco small subunit was fused to the non-plant derived *crtI* and *crtW*, while *psy* naturally contains its plastid transit peptide.

### *Nicotiana benthamiana* allowed for the rapid functional analysis of carotenogenic genes involved in ketocarotenoid biosynthesis

*Nicotiana benthamiana* is a well-established biofactory system for transient expression, characterized by large leaves that facilitate infiltration and rapid production of recombinant proteins. This species was selected due to its natural ability to supply the precursor GGPP and carotenoids, which support the synthesis of ketocarotenoids. In order to assess the effect of expressing the heterologous genes *psy*, *crtI* and *crtW*, we performed transient expression experiments using Agrobacteria-mediated delivery in fully developed leaves. Since mature chloroplasts in these leaves naturally synthesize carotenes and xanthophylls, we aimed to determine if the introduced genes could extend the pathway and produce ketocarotenoids, thereby altering the carotenoid composition. Furthermore, we investigated whether endogenous enzymes, in conjunction with the β-carotene ketolase would be sufficient for ketocarotenoid production. We tested single-gene constructs (pY; pI; pW) and multi-combinatorial (pY + pI; pY + pW; pI + pW; pY + pI + pW).

High-performance liquid chromatography (HPLC) analysis of ethanolic leaf extracts was performed to qualitatively evaluate the carotenoid profile, with each leaf serving as an independent biological replicate. Based on retention time (Figure S1), absorption spectra, and comparison with available standards, smaller amounts of neoxanthin (Neo), violaxanthin (Vio) and β-carotene (β-car) were identified. To aid pigment identification, we analyzed ethanolic extracts of *Arabidopsis thaliana* and *Nicotiana tabacum* leaves under the same HPLC conditions and compared the retention times and absorption spectra to literature reports, including those from *Phaeodactylum tricornutum*^34^. In *P. tricornutum*, chlorophyll elutes at around 11 min and displays characteristic absorption maxima at 445 and 663 nm. In our Arabidopsis samples (Figure S2), peaks at 13.4 and 14.4 min showed similar dual maxima (457.1/646.3 nm and 430.4/662.3 nm, respectively), consistent with chlorophyll^35^. Additionally, a peak at 8.9 min had identical absorption maxima (447.4 and 475.3 nm) to a lutein standard, confirming its identity. However, under our HPLC conditions, we could not definitively discard the co-elution of lutein and its isomer zeaxanthin. Based on this comparison, along with information on the different polarities of the pigment molecules and available carotenoid descriptions in literature reports (e.g^16,36,37^), we identified putative zeaxanthin/lutein (*Z/L), chlorophyll b (*Chl b) and chlorophyll a (*Chl a). Trace amounts of two carotenoids (peak 1 and 2, Figure S1) were detected exclusively in the leaves infiltrated with the *crtW* gene. While the unidentified pigment eluted at ~ 10 min (peak 3) was detected in all the samples, it was found to be present in higher amounts in the pW-infiltrated leaves. None of these peaks corresponded to free astaxanthin, even though the leaves had an orange-brown coloration compared to other leaves (e.g. negative control, pY-infiltrated leaves). However, further analysis would be necessary to confirm the structure of these compounds. The ethanolic extracts could contain ketocarotenoid intermediates since astaxanthin biosynthesis requires four enzymatic steps from β-carotene, divided into two different pathways. Alternatively, the peaks could correspond to astaxanthin fatty acid mono- and/or di-esters^38^which are less polar and thus exhibit higher retention times. With these results, we proceeded with the transformation of plant cell cultures using these constructs.

### Elicitation experiments of carotenoid synthesis in undifferentiated non-photosynthetic BY-2 cells

To investigate the effects of *crtW* in non-green tissues characterized by rapid growth and controlled conditions, tobacco BY-2 cultured cells were selected as a potential platform for ketocarotenoid production. Previous studies have shown that BY-2 cells grown in darkness do not accumulate specialized carotenoids^39,40^including key precursors for ketocarotenoids biosynthesis. Since carotenoids play a role in photoprotection, light exposure was hypothesized to induce carotenoid biosynthesis. Consequently, BY-2 cells were transferred into liquid MS medium with reduced sucrose (2% (w/v)) and grown under controlled light conditions. Cell suspension cultures were refreshed by transferring 3% (v/v) of cells into 25 ml of MS medium, with a stepwise 50% reduction in sucrose content (Table S1), following the methodology outlined by Dubreuil and colleagues^41^. This approach, previously applied to Arabidopsis, promoted the differentiation of chloroplasts from proplastids. We also tested various kinetin concentrations (0 to 27.88 µM), since this artificial cytokinin is known to aid proplastid differentiation^42^. We aimed to identify optimal conditions for enhancing carotenoid biosynthesis while maintaining cell viability. Following a preliminary analysis of BY-2 cell viability and total carotenoid content, specific samples were collected and subjected to HPLC analysis to assess the new carotenoid profile in BY-2 cells.

After four months, carotenoid production was observed under optimal growth conditions. HPLC analysis of specific samples (Figure S3) revealed the presence of at least three carotenoids, identified as neoxanthin, violaxanthin and β-carotene, based on spectral properties and co-chromatography with standards. These were detected in cultures grown with 3% sucrose and 9.29 µM of kinetin, 1% sucrose and 13.94 µM of kinetin, and 0.5% sucrose and 18.59 µM of kinetin. As high kinetin concentrations and lower sucrose concentrations reduced cell division, we lowered kinetin concentrations for better cell viability. After approximately one year, BY-2 cells grown under a photoperiod exhibited the capability to produce carotenes and xanthophylls (Figure S3), which were absent in the WT cells grown in the dark. Moreover, chlorophyll was not detected throughout the experiment.

### Combinatorial nuclear transformation of BY-2 cells generated distinct phenotypes

**Fig. 3:**
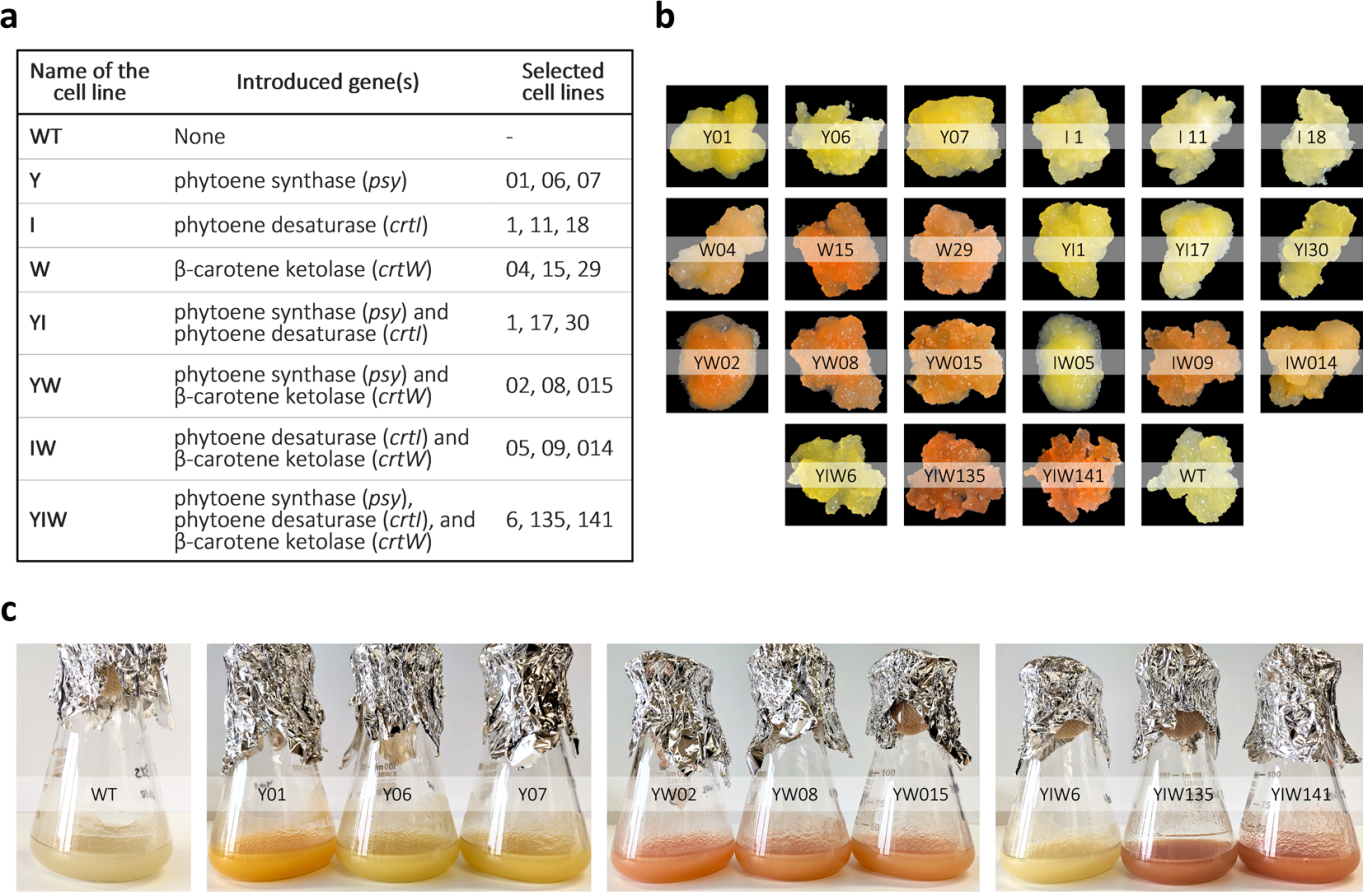
Phenotype of *Nicotiana tabacum* BY-2 cell lines compared to the wild-type. **(a)** Classification of BY-2 cell lines containing the heterologous *psy*, *crtI*, and/or *crtW* genes resulting from the single and combinatorial transformations with the pY, pI, and/or pW vectors. **(b)** Pale, yellow or pink-orange pigmented tobacco BY-2 *calli*. **(c)** BY-2 cultured cells, the WT and the highest yielding cell lines for the production of xanthophylls, astaxanthin, and canthaxanthin.

The carotenoid pathway of BY-2 cells was metabolically engineered through combinatorial transformation with carotenogenic genes, all under the control of the strong promoter CaMV 35 S. Seven independent transformation events were conducted (Table S2), involving the individual transformation of BY-2 cultured cells with *phytoene synthase* (*psy*), *phytoene desaturase* (*crtI*), or *β-carotene ketolase* (*crtW*) genes from *Zea mays*, *Pantoea ananatis*, and *Brevundimonas* sp. SD212, respectively. Combinatorial transformations involved two genes in the following combinations: *psy* with *crtI*, *psy* with *crtW*, and *crtI* with *crtW*. A final multigene transformation included all three genes. For the identification of these cell lines, we used the last letter of each gene introduced to name the cell line, as follows: “Y” represents cells with the heterologous *psy* gene, “I” represents *crtI*, and “W” represents *crtW*. When two or more genes were combined, the letters were combined accordingly: “YI” refers to cell lines with both *psy* and *crtI*, “YW” refers to cells with *psy* and *crtW*, “IW” refers to cell lines with both *crtI* and *psy*, and “YIW” designates cells containing all three genes, *psy*, *crtI*, and *crtW*. These names (Y, I, W, YI, YW, IW, and YIW) will be used throughout the text to refer to each specific transformation event (Fig. 3a). The numbers following these letters indicate the specific *calli* selected. To target the bacterial CRTI and CRTW enzymes to the plastids, a transit peptide from the pea RuBisco small subunit was included in the expression cassettes. This transit peptide has been successfully used in various systems, including in rice endosperm, maize, and soybean^16,37,43^. The maize *psy* gene contains an endogenous transit peptide and was used without modifications. Numerous *calli* were generated from each transformation and underwent several rounds of antibiotic selection before the screening.

The newly established transgenic BY-2 lines showed distinct phenotypes among the different transformation events and compared to pale-yellow wild-type *calli*. *Calli* transformed with *crtI* resembled the wild-type, whereas those transformed with *psy* displayed light to dark yellow pigmentation. In contrast, *calli* transformed with the *crtW* gene exhibited light brownish, salmon, or orange coloration (Fig. 3b). Co-expression of two or three genes produced various colorations: white or yellow (transformation with constructs pY + pI), white or pink-orange (transformation with constructs pY + pW or with pI + pW) and white, yellow or pink-orange colored *calli* (transformation with constructs pY + pI + pW). Despite these color changes, all the transgenic BY-2 lines exhibited normal growth and morphology similar to wild-type *calli*. The intensity of pigmentation in transgenic *calli* increased after three weeks of growth under controlled light conditions. Several *calli* from each transformation were selected based on color. No modifications to the culture medium, such as adjustments in sucrose or kinetin concentrations, were made, as the *calli* exhibit color upon light exposure post-transformation. However, kinetin potential as an elicitor to enhance pigment production could be explored in future studies.

The presence of transgenes was confirmed by PCR using specific primers, and no bands corresponding to *psy*, *crtI* or *crtW* were detected in the wild-type (negative control) (Figure S4). Following this confirmation, we established cell suspension cultures. A total of twenty-one independent lines were selected, and liquid cultures were established (Fig. 3c, Figure S5). Importantly, expression of the heterologous genes did not affect growth rate or biomass production compared to WT cell suspension culture. Both WT and transgenic BY-2 *calli* were subcultured every three weeks, and cell suspensions were subcultured on the 7th day of the growth curve.

### Engineering of the carotenoid biosynthetic pathway led to the production and accumulation of ketocarotenoids

**Fig. 4:**
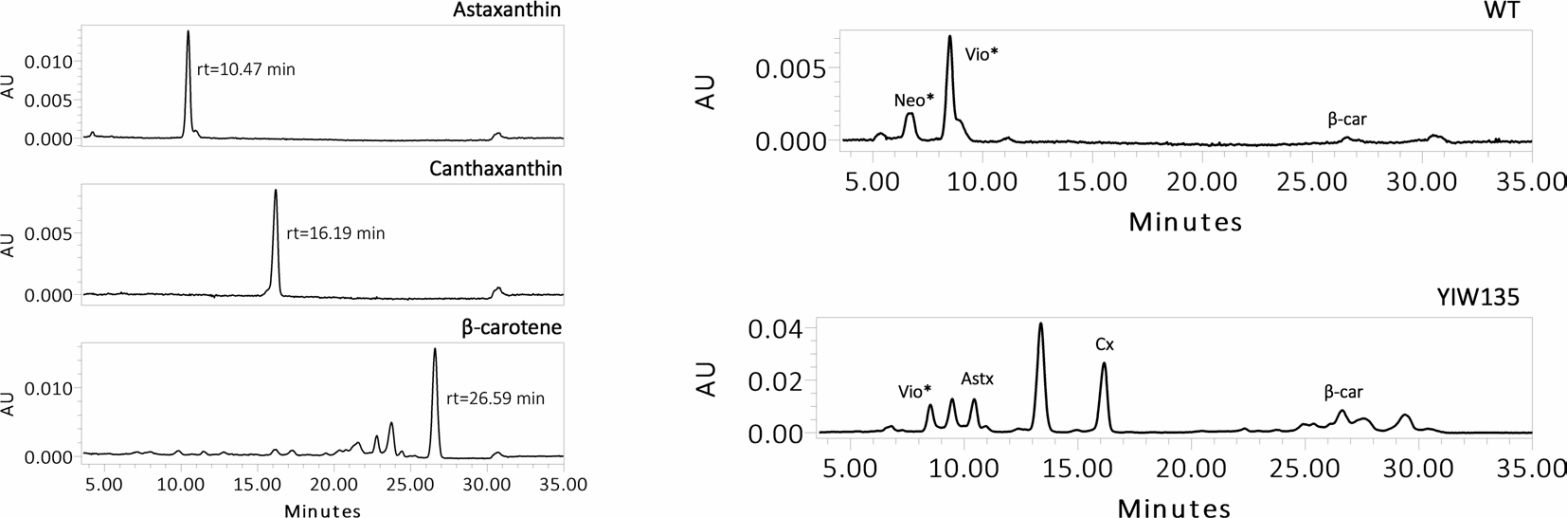
Carotenoid profile of tobacco BY-2 wild-type (WT) and the highest ketocarotenoid-producing line (YIW135). The resulting chromatograms were recorded at a wavelength of 445 nm, with the y-axis scaled to the highest peak to enhance clarity. The carotenoids identified were putative neoxanthin (*Neo), putative violaxanthin (*Vio), astaxanthin (Astx), canthaxanthin (Cx) and β-carotene (β-car).

To determine the carotenoid composition of WT and transgenic BY-2 cell lines, cells were harvested on the 7th day of the growth curve. Pigment analysis was performed at this phase, because a progressive increase in color intensity was observed throughout the growth period. HPLC analysis revealed a correlation between the observed coloration and the resultant carotenoid profile. Wild-type BY-2 cells accumulated β-carotene and a mixture of xanthophylls, including putative neoxanthin and violaxanthin (Fig. 4, Figure S6). Their identification was based on comparisons with chromatographic and spectral data obtained in previous analysis of Arabidopsis and BY-2 extracts (Figure S2, S3). Collectively, this information allowed for the qualitative identification of these two xanthophylls without relying on standards. As expected, no astaxanthin or canthaxanthin were detected in the WT extracts.

The tobacco lines I, Y and YI exhibited similar pigment compositions, featuring putative neoxanthin, violaxanthin and β-carotene as the predominant pigments. Although these lines shared a similar carotenoid profile, their colors varied due to differences in the accumulation of each pigment. The cell lines Y displayed a bright lemon-yellow color, likely reflecting a higher accumulation of xanthophylls, while the cell lines YI showed a darker yellow coloration. Moreover, these lines exhibited an increased number of compounds eluting between 17 and 25 min in contrast to the cell lines I. These compounds could potentially be violaxanthin fatty acid esters, which were also identified in *Nicotiana glauca* leaves^36^. The xanthophylls zeaxanthin and lutein, isomers present in *N. tabacum* leaves^44^are challenging to distinguish due to a structural difference involving a double bond within one of the ionone rings (ε-ring). Co-chromatography with a lutein standard confirmed its absence in tobacco BY-2 lines. Although HPLC analysis with a zeaxanthin standard was not performed, the synthesis of zeaxanthin in BY-2 cells is supported by the presence of its precursor, β-carotene, and its derivative, violaxanthin.

The introduction of the β-carotene ketolase enzyme (CRTW) completed the carotenoid biosynthetic pathway, resulting in the production of ketocarotenoids. Cell lines W, expressing the *crtW* gene, exhibited distinct carotenoid profiles compared to the wild-type, and all produced both astaxanthin and canthaxanthin, with several additional compounds detected. For the HPLC analysis, the total carotenoid extract was separated using a C18 column, which is a reverse phase column with a non-polar stationary phase. Due to the elution conditions employed in this methodology, more polar compounds (such as xanthophylls) eluted earlier than non-polar compounds (carotenes)^45–47^and not all substrates are converted into the final product of the pathway (astaxanthin).

**Fig. 5:**
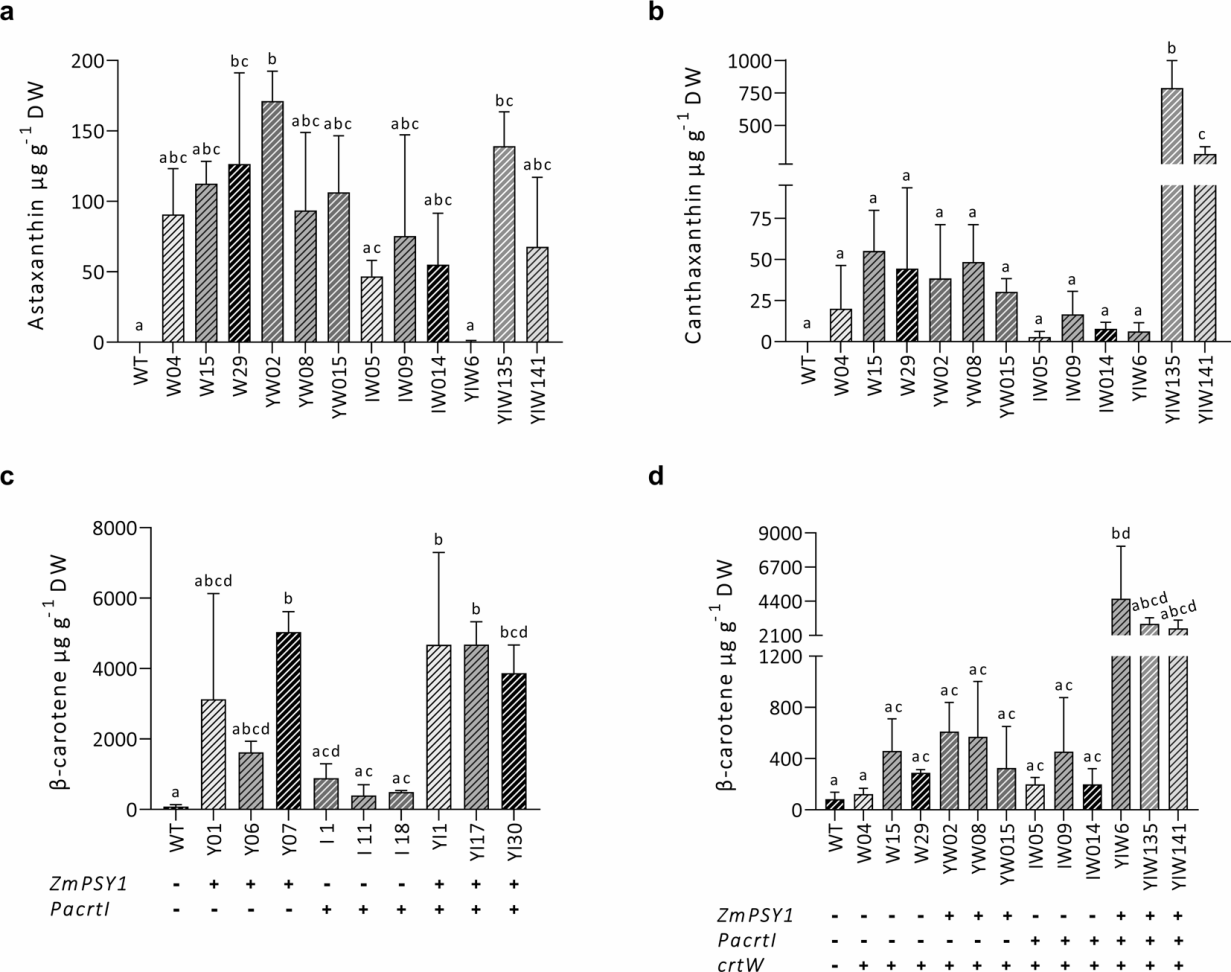
Carotenoid content in tobacco BY-2 cell lines is shown as µg g^−1^ DW (micrograms per gram of dry weight). Quantification of **(a)** astaxanthin and **(b)** canthaxanthin in ketocarotenoid producing cell lines. Quantification of β-carotene in **(c)** xanthophyll and **(d)** ketocarotenoid producing cell lines. The presence (+) or absence (-) of the heterologous genes is indicated for each line. Each data point represents the mean result derived from three biological replicates from the HPLC run, with error bars indicating the standard deviation (SD) for each measurement. Different letters above the bars indicate statistically significant differences (*p* value (p) < 0.05), determined by one-way ANOVA followed by Tukey’s test with a single pooled variance.

Quantitative data showing the levels of astaxanthin, canthaxanthin and β-carotene in all cell lines is summarized in Table 1. A notable increase in β-carotene levels was observed in cell lines expressing *psy*, either singly or in combination with *crtI* gene. In the transgenic lines Y and YI, β-carotene levels increased 20- to 60-fold compared to wild-type cells. In the cell lines YIW, co-expressing all three genes resulted in an increase in β-carotene up to 55-fold. Transformation with the *crtW* gene alone enabled BY-2 cells to produce ketocarotenoids (Figure S6), using naturally synthesized xanthophylls as substrates. The astaxanthin content in the cell lines W ranged from 90 to 127 µg g^−1^ DW, while canthaxanthin amounts ranged from 20 to 55 µg g^−1^ DW (Table 1; Fig. 5). To evaluate the enhancement of precursor supply and putative increased ketocarotenoid yield, lines co-expressing *crtW* with *psy* and *crtI* were analyzed. The optimal combination for astaxanthin production was achieved through the co-expression of *crtW* with *psy*. In comparison to cell line W04, YW02 exhibited a 1.9-fold increase in astaxanthin and a 2.4-fold increase in canthaxanthin accumulation. Conversely, co-expression of *crtW* with *crtI* (lines IW) resulted in decreased ketocarotenoid levels. Preliminary semiquantitative PCR analysis suggested that the expression of endogenous genes such as *phytoene synthase* was higher in the engineered cell lines when comparing to the WT (Figure S7). However, heterologous gene expression varied between the different gene combinations used, potentially contributing to differences in carotenoid yields. In some cases, gene expression did not directly correlate with pigment accumulation. For example, cell line I1 exhibited higher expression of both endogenous *phytoene desaturase* and heterologous *crtI* but accumulated only low levels of β-carotene (Table 1), whereas line IW09 showed low expression of heterologous *crtI* and *crtW* genes, consistent with its reduced ketocarotenoid content. Notably, co-expression of all three genes (lines YIW) led to a significant increase in canthaxanthin production. The highest accumulation of canthaxanthin (788 and 279 µg g⁻¹ DW) was observed in line YIW135 and YIW141, a 39- and 14-fold increase relative to the single *crtW* line (W04). These results indicate that engineering upstream pathway (via *psy* and *crtI*) boosts precursor supply, favoring high-level production of ketocarotenoids in non-photosynthetic BY-2 cells.

Among the population of transgenic BY-2 lines examined, one line stood out. Cell culture YIW6 displayed a yellow color. PCR analysis of the genomic DNA confirmed the presence of the *crtW* gene in YIW6 cells. However, the carotenoid profile obtained by HPLC showed only trace amounts of ketocarotenoids, indicating that the conversion of β-carotene and zeaxanthin was limited, resulting in an accumulation of β-carotene. Astaxanthin and canthaxanthin levels were merely 0.5 and 6.2 µg g^−1^ DW, respectively. This observation was consistent with the low transcript levels of *crtW* detected in the semiquantitative PCR analysis. The expression of the endogenous *psy* and *pds*, as well as the heterologous *crtI* gene, and the production of β-carotene were higher than those observed in the cell line YIW135, indicating the possibility of precursor accumulation without efficient conversion into ketocarotenoids. The expression of *crtW* in YIW6 was detectable only after 40 PCR cycles using undiluted cDNA (Figure S8), reflecting minimal transcript abundance. Further studies are necessary to investigate whether the variability in ketocarotenoid accumulation among the different cell lines is attributed to factors such as transgene copy number, insertion site, transcriptional-level silencing, or enzyme efficiency.

**Table 1:** Carotenoid content in wild-type and 21 cell lines. Carotenoid levels are represented as µg g^−1^ DW and expressed as a percentage of total carotenoid extract (% TCE)^*^. Each value represents the mean of three biological replicates ± SD. Statistical differences between groups were evaluated using ordinary one-way ANOVA followed by Tukey’s multiple comparisons test (single pooled variance). Groups not sharing the same letter are significantly different at *p* < 0.05. nd: not detected.

	Astaxanthin		Canthaxanthin		β-carotene	
	µg g^−1^ DW	% of TCE	µg g^−1^ DW	% of TCE	µg g^−1^ DW	% of TCE
**Wild-type**	nd	-	nd	-	83.6^a^ ± 53.4	2.6 ± 0.9
**Y01**	nd	-	nd	-	3 128.5^abcd^ ± 3 001.2	6.0 ± 5.2
**Y06**	nd	-	nd	-	1 624.2^abcd^ ± 315.5	20.7 ± 4.2
**Y07**	nd	-	nd	-	5 035.8^b^ ± 578.8	26.0 ± 2.5
**I1**	nd	-	nd	-	891.6^acd^ ± 408.1	12.1 ± 2.7
**I11**	nd	-	nd	-	395.7^ac^ ± 309.7	12.6 ± 8.0
**I18**	nd	-	nd	-	496.1^ac^ ± 42.1	10.9 ± 0.9
**W04**	90.8^abc^ ± 32.4	15.5 ± 2.2	20.0^a^ ± 26.3	1.4 ± 1.2	124.2^a^ ± 45.2	1.0 ± 0.4
**W15**	112.6^abc^ ± 15.8	16.3 ± 2.5	55.1^a^ ± 24.7	2.8 ± 1.3	460.9^ac^ ± 249.5	2.9 ± 1.3
**W29**	126.5^bc^ ± 64.8	22.9 ± 3.2	44.5^a^ ± 49.1	2.5 ± 1.6	289.2^ac^ ± 25.0	2.6 ± 1.5
**YI1**	nd	-	nd	-	4 675.9^b^ ± 2 624.0	33.4 ± 1.9
**YI17**	nd	-	nd	-	4 685.5^b^ ± 642.2	36.3 ± 1.5
**YI30**	nd	-	nd	-	3 872.9^bcd^ ± 797.3	30.1 ± 3.7
**YW02**	171.3^b^ ± 21.2	14.8 ± 3.6	38.4^a^ ± 32.7	1.6 ± 1.7	611.5^ac^ ± 227.9	3.0 ± 1.1
**YW08**	93.5^abc^ ± 55.5	11.3 ± 0.4	48.5^a^ ± 22.7	2.1 ± 0.2	570.6^ac^ ± 431.7	3.1 ± 2.0
**YW015**	106.3^abc^ ± 40.3	15.2 ± 1.4	30.4^a^ ± 7.9	1.7 ± 0.5	326.8^ac^ ± 323.9	1.8 ± 1.2
**IW05**	46.7^ac^ ± 11.4	11.9 ± 1.0	2.8^a^ ± 3.3	0.4 ± 0.3	199.7^ac^ ± 53.3	2.1 ± 1.1
**IW09**	75.4^abc^ ± 71.9	12.2 ± 2.3	16.7^a^ ± 13.9	1.3 ± 0.6	455.1^ac^ ± 421.9	2.1 ± 1.2
**IW014**	55.0^abc^ ± 36.6	19.6 ± 0.6	7.8^a^ ± 4.1	1.3 ± 0.1	199.0^ac^ ± 122.1	1.7 ± 1.2
**YIW6**	0.5^a^ ± 0.8	0.2 ± 0.4	6.2^a^ ± 5.3	0.3 ± 0.3	4 584.0^bd^ ± 3 517.8	31.9 ± 7.1
**YIW135**	139.3^bc^ ± 24.3	7.1 ± 0.3	787.9^b^ ± 213.3	12.9 ± 2.3	2 878.4^abcd^ ± 413.8	8.0 ± 1.3
**YIW141**	67.8^abc^ ± 49.3	5.4 ± 0.2	279.0^c^ ± 56.9	5.9 ± 0.7	2 578.9^abcd^ ± 551.9	9.5 ± 0.3

^*^ The sum of the integrated area of each peak corresponds to the total carotenoid content for each sample. To determine the percentage of each specific carotenoid (astaxanthin, canthaxanthin, or β-carotene) within the total extract, the area of each peak was divided by the total peak area.

## Discussion

The growing market demand for carotenoids in various industries reflects a consumer preference for natural, health-promoting compounds. For this reason, there is a recognized need to improve and increase carotenoid production. This cannot be achieved from natural sources due to the drawbacks of natural production, such as high costs and the complexity of isolating specific carotenoids, namely ketocarotenoids. Therefore, alternative biological production systems are being explored to achieve cost-effective ketocarotenoid synthesis, prioritizing environmental friendliness and safety for human use. In this context, efforts have been made to improve natural large-scale carotenoid production using heterologous systems, including overexpression of rate-limiting enzymes, downregulation of competing pathways, and enhancement of carotenoid storage capacity^48^.

### Unveiling ketocarotenoid production in non-photosynthetic undifferentiated cells

Within the various expression systems available, plant cell suspension cultures have already demonstrated the potential for commercial production of high-value molecules. This is exemplified by the agreement between Protalix and Pfizer to produce Elelyso^®^, a therapeutic enzyme for the treatment of Gaucher disease^49^using cultured carrot cells. Beyond recombinant proteins, the production of pigments in plant cell cultures has also been reported. Carrot, tomato, and blueberry cultured cells have been shown to naturally accumulate pigments^50–52^but few studies have reported heterologous production. Examples include tobacco undifferentiated cells producing anthocyanins^53^and BY-2 cells producing betalain, betanidin, betanin and amaranthin^32,33^.

In this report, we show that BY-2 cells hold potential for producing secondary metabolites, in particular ketocarotenoids that are not naturally accumulated in these cells. We show that undifferentiated cells are able to easily adapt to the heterologous production of small metabolites including those typically associated with photosynthesis, a process that is not observed in these systems. As the establishment of this cell line occurred many decades ago, they do not naturally produce and store specific photosynthesis-related secondary metabolites, such as specialized carotenoids, which are normally involved in photoprotection. In higher plants, light is known to influence the regulation of carotenoid biosynthesis and the expression of carotenogenic genes^54^. Typically, tobacco BY-2 cells grow in the dark, in MS medium supplemented with a carbon source, vitamins and auxins. In our study, prior to the insertion of foreign carotenogenic genes, BY-2 cell cultures were transitioned from total darkness to a 16-hour light period, resulting in the production of carotenes and xanthophylls.

Using undifferentiated cells in culture offers the advantage of high proliferation rates and the ability to adapt to various genetic and environmental modifications, making them ideal for scalable production. Compared to other expression systems, BY-2 cells offer advantages such as fast growth, controlled and confined environment, reduced costs, adherence to good manufacturing practices (GMP) and safety^55,56^.

### Strategy for ketocarotenoid production in BY-2 cells

Our strategy involved the combinatorial expression of carotenogenic genes controlled by a strong promoter. Since BY-2 cells accumulate low amounts of carotenoids, we hypothesized that enhancing the upstream components of the ketocarotenoid pathway would increase overall production of carotenoids, as shown in other studies. In maize and rice^16,43,57,58^the heterologous expression of *phytoene synthase* and *desaturase* genes led to increased production of lycopene and β-carotene, mediated by the plant endogenous lycopene β-cyclase enzyme. These enzymes, though derived from different organisms, perform identical functions as the native enzymes^59^.

In BY-2 expressing the *crtI* gene, a small increase in total carotenoid content was observed, while maintaining a carotenoid profile (β-carotene, neoxanthin and violaxanthin) similar to wild-type cells, as described by Schaub and colleagues^40^. Moreover, these authors reported that expression levels of several genes varied between transgenic and wild-type cultures. In their study, the introduction of *crtI* did not consistently affect gene expression^60,61^and this variability was likely a consequence of growth differences among the transgenic cultures rather than a direct effect of *crtI*^40^. Interestingly, our cell lines Y and YI exhibited a yellow coloration, with Y displaying a particularly strong phenotype due to substantial β-carotene accumulation. Semiquantitative PCR preliminary data from the Y07 and YI17 transgenic lines showed a higher expression of tobacco (endogenous) and maize (heterologous) *psy* genes, correlating with the observed yellow pigmentation and β-carotene accumulation. But, for YI17 a very low expression of *pds* and *crtI* genes was observed compared to cell line I1. This suggests that *crtI* expression only slightly impacts carotenoid biosynthesis, with the PSY enzyme playing a critical role in expanding the ketocarotenoid precursor pool. As previously reported by Cazzonelli and Pogson, phytoene synthase is not only a key regulatory enzyme but also represents a rate-limiting step in carotenogenesis^59^. Moreover, carotenoid accumulation is influenced not only by transcript abundance but also by factors such as enzyme kinetics, protein stability, and subcellular compartmentalization.

Despite the initially low accumulation of carotenoids in BY-2 wild-type cells, the expression of the single *crtW* gene enabled the production of astaxanthin and canthaxanthin and significantly enhanced total carotenoid accumulation. This was attributed to the increased metabolic activity and up-regulation of endogenous carotenogenic metabolites. Thus, the introduction of this missing enzyme successfully completed the ketocarotenoid pathway. Transformation with *crtI* or *psy* combined with *crtW* gene produced a range of colorful cell lines, likely due to strong expression of the introduced genes. The IW cell lines exhibited lower levels of astaxanthin and canthaxanthin compared to the cell lines W, likely due to the diversion of the carotenoid pathway toward xanthophyll synthesis by β-carotene hydroxylase (CHYB) enzyme. Although neoxanthin and violaxanthin were not quantified, the chromatogram of the cell line IW09 (Figure S6) suggests higher levels of these xanthophylls than in line W04 or W15. The reduced accumulation of ketocarotenoids in IW09 was accompanied by lower expression levels of all analyzed genes relative to the respective controls, except for the endogenous *psy* gene. Our multi-enzyme cell lines were designed to enhance product yield and regulate precursor flux. Remarkably, cell lines YIW135 and YIW141 exhibited the highest accumulation of canthaxanthin, despite *crtW* transcript levels being lower in YIW135 compared to W15. Canthaxanthin is naturally found in low amounts, making it challenging to obtain. The production of higher levels of this pigment is particularly significant in our study and the ability to generate cell lines with elevated canthaxanthin levels represents an achievement and addresses a specific interest in our research. All lines co-expressing *crtW* with additional heterologous genes show reduced *crtW* expression levels compared to W15. This suggests that *crtW* expression may be negatively affected when co-expressed with other genes. Besides YIW6, the lowest *crtW* level is observed in YW02, which also exhibits very low expression of the heterologous *psy* gene compared to the Y07 cell line. Interestingly, despite this, YW02 produces more astaxanthin than the W04, W15, or W29 lines. This further supports the observation that transcript abundance is not necessarily proportional to carotenoid yield. The distinct phenotype of cell line YIW6, with lower ketocarotenoid amount, could be attributed to low *crtW* expression (Figure S7), enzyme kinetics, gene copy number and insertion site. A similar low ketocarotenoid accumulation was observed in *Lilium* leaves, with only 200 ng of astaxanthin per gram of fresh material^62^. Azadi and colleagues hypothesized that the low amount, like our findings in YIW6, was due to the low expression of *crtW*. The semiquantitative PCR results provide a preliminary indication of relative changes across metabolic engineered cell lines. However, carotenoid accumulation depends on multiple factors beyond transcript abundance, including precursor supply, metabolic flux through upstream pathways like MVA and MEP, and post-transcriptional regulation. Since these BY-2 cells were able to adapt to accommodate an active secondary metabolism, understanding these complexities requires a broader view. Therefore, future whole transcriptomic analyses will aid the study of regulatory changes affecting carotenoid biosynthesis and related metabolic networks, which is essential for fully interpreting pigment accumulation in these engineered lines.

### Understanding key factors in ketocarotenoid production

Several factors must be taken into account when planning heterologous production of ketocarotenoids. The choice of *β-carotene ketolase* gene source used for transformation can impact ketocarotenoid production. Potential differences in efficiency between marine bacterial (CRTW) and algal (BKT) ketolases have been reported^13,20,63,64^. Other factors to consider include the availability of precursors, and the expression of other enzymes that boost ketocarotenoid precursor production.

In microbial systems, Fraser and colleagues performed an in vitro characterization of marine bacterial, algae and terrestrial bacterial enzymes, including β-carotene hydroxylase (CRTZ) and ketolase (CRTW and BKT). They demonstrated that marine bacterial CRTZ converted canthaxanthin to astaxanthin preferentially, whereas *Erwinia* CRTZ favored zeaxanthin formation from β-carotene. CRTW/BKT enzymes responded readily to fluctuations in substrate levels^65^. The complex conditions of marine environments, including high salinity, pressure, low temperature, and specialized lighting conditions, have prompted marine microorganisms to evolve enzyme systems that are more stable and active than those found in plants or animals. These challenges have resulted in notable distinctions between marine microbial enzymes and their terrestrial counterparts^66^. Understanding the interplay between *crtW* expression, host plant systems, and carotenoid profiles is essential, as *β-carotene ketolase* expression in different species has differential effects on the overall carotenoid accumulation. Variability in ketocarotenoid production may arise from transformation methods, specific genes and promoters employed, plant species, or tissue type. Coordinating these factors is crucial to attain optimal ketocarotenoid production within the selected system.

The heterologous production of astaxanthin and canthaxanthin in plants, through nuclear or plastidial modification, is well documented. Huang and colleagues expressed algal *β-carotene ketolase* and hydroxylase genes in tomato, resulting in ketocarotenoid accumulation (Astx = 3.12 mg g^−1^ DW)^67^. Further investigations introduced combinations of *β-carotene hydroxylase* and *ketolase (crtZ* and *crtW)* genes into various plants, including *Brassica napus* (Astx = 0.6 µg g^−1^ DW), *Solanum tuberosum* (Astx = 0.51 µg g^−1^ DW), *N. benthamiana* (Astx = 14.7 µg g^−1^ DW), and maize (0.016 mg g^−1^ DW). Soybean seeds, transformed with *phytoene synthase* and *crtW*/*bkt* genes, also yielded ketocarotenoid-rich transgenic seeds (Astx = 7 µg g^−1^ DW)^12,37,68–70^. Other examples are outlined in Table S3. Chloroplast genome engineering enables efficient, targeted gene insertion, and robust foreign gene expression without epigenetic changes^20,71^. Successful cases include astaxanthin production in *N. tabacum* (5.44 mg g^−1^ DW) and *Lactuca sativa* (178 µg g^−1^ fresh weight) via plastidial transformation^63,72^.

Ketocarotenoid production has also been achieved in microalgae and fermentation systems, with several successful cases (reviewed in^73^) although there are a few associated drawbacks. For example, microalgal species frequently exhibit a slow growth rate, leading to an elevated risk of contamination during large-scale cultivation and incurring high production costs^25^. While bacterial systems are fast-growing and economical, they typically require the introduction of multiple heterologous genes to engineer the entire carotenoid biosynthetic pathway, along with careful optimization of precursor supply and availability. Yeast heterologous expression introduces challenges in protein translation and folding within a non-natural environment. Furthermore, this process relies on using metabolic intermediates produced by the host’s enzymatic machinery. In multistep pathways, efficiency may be compromised by potential losses of intermediates through diffusion, degradation, or conversion by competing enzymes^74^. In contrast, our BY-2 cell-based system contains an endogenous carotenoid pathway. The resulting cell lines accumulate quantifiable levels of ketocarotenoids through stable integration of heterologous genes and exhibit a spectrum of distinct colors, reflecting unique combinations of carotenoid composition. The main achievement of this work is the demonstration that undifferentiated, non-photosynthetic plant cultured cells can support multi-gene engineering strategies to produce pigments reliably. This work introduces the innovative concept of plant cell suspension cultures as flexible and sustainable biofactories, with potential for producing a wide range of specialized molecules beyond ketocarotenoids.

### Future prospects in ketocarotenoid research: paving the way for industrial-scale ketocarotenoid manufacturing

The study of ketocarotenoid pathways is relevant for understanding intrinsic cellular mechanisms, along with applications in biotechnology. A key aspect is the subcellular localization of ketocarotenoids in these BY-2 cells, particularly the ability to compartmentalize secondary metabolites within cellular structures not available in yeast or bacteria. Although still under investigation, this localization is likely to be plastidial structures, involving differentiation from proplastids upon specific triggers^19,75^. This sub-compartmentalization is a major advantage compared with microbial platforms and opens the way to the engineering of vesicles or organelles in other systems such as yeasts. However, this is beyond the scope of the present study and will be addressed in future research. Recent advances in synthetic biology, metabolic engineering, and systems biology have opened new ways for production of secondary metabolites, shifting from extraction from natural sources into engineered biofactories. Although microbial production is a viable option, most studies employed extensive genetic engineering strategies. In contrast, the plant cell cultures presented in this study offer a potential alternative. Due to their high metabolic rates, driven by rapid cell mass proliferation and homogeneity^39,76^plant cell cultures enable the study of biosynthetic pathways and the formation of secondary metabolites within a short cultivation time, typically around 1–4 weeks. To the best of our knowledge, our line of research towards consistent ketocarotenoid production in non-photosynthetic undifferentiated cell cultures is pioneering. In the future we will validate the scalability of our system, with ongoing efforts focused on establishing pilot-scale cultures. Precision fermentation and controlled metabolic flux will improve yields, reduce costs, and address regulatory challenges. Efforts to expand substrate flexibility, such as utilizing agricultural residues, can also enhance economic feasibility and environmental sustainability of production processes. Overall, the future of industrial-scale ketocarotenoid manufacturing lies in integrating AI-driven design with advanced bioreactor systems for predictive optimization and real-time production monitoring.

To conclude, *Nicotiana tabacum* BY-2 cells are a highly versatile platform with broad applications in Molecular Farming, including the production of high-value metabolites such as ketocarotenoids. When this cell line was first established in the 1970’s^77^its future utility in biotechnology was unforeseen. Decades later, BY-2 cells have demonstrated remarkable plasticity and adaptability, thus establishing them as a robust system for metabolic engineering, recombinant protein production, and secondary metabolite biosynthesis. The rapid growth of these cells, along with their ease of genetic modification and scalability, makes BY-2 cells an appealing candidate for use as a production chassis. As the demand for sustainable and controlled bioproduction systems continues to grow, the role of BY-2 cells is set to become increasingly important in the advancement of plant-based biofactories.

## Experimental procedures

### Plant material

#### Tobacco BY-2 cell culture conditions

*Nicotiana tabacum* cv. Bright Yellow 2 (BY-2) cells were maintained as described by Rebelo and colleagues^56^. Briefly, cultures were grown in supplemented Murashige and Skoog (MS, Duchefa) medium containing 3% (w/v) of sucrose (Duchefa), inositol, phosphate, thiamine, and 2,4-Dichlorophenoxyacetic acid), on an orbital shaker (OVAN I10-O + ACOP. E) at 28 °C and 120 rpm in darkness. Stock cultures were maintained as *calli* on MS medium solidified with 0.7% agar (Duchefa), also kept in darkness at 28 °C.

To prepare BY-2 cells for transformation with carotenogenic genes, cultures were grown under a 16-hour light and 8-hour dark photoperiod, with a LED light intensity of 80–100 µmol m^−2^ s^−1^. Cell suspension cultures were renewed by transferring 3% (v/v) of the culture into 25 ml of MS medium, gradually reducing sucrose concentrations: starting at 3%, then 2%, 1%, and finally 0.5%. Simultaneously, sterilized kinetin (Duchefa) was added to the medium at concentrations ranging from 0.46 to 27.88 µM. Biomass was collected at various stages of the elicitation experiment and stored at −80 °C for further analysis.

#### *Growth conditions ofNicotiana benthamianaplants*

*Nicotiana benthamiana* plants used for transient expression were grown for six weeks in a controlled chamber (Fitoclima 4600, Aralab) at 22 °C, with an 8-hour light and 16-hour dark photoperiod. The light intensity was maintained at 80–100 µmol m^−2^ s^−1^, and relative humidity was set to 65% to ensure optimal growth conditions.

### Vector construction

#### *Phytoene synthase(psy) andphytoene desaturase(crtI)*

Plasmids p326-ZmPSY1 and pHORP-PacrtI^43^ (kindly offered by Changfu Zhu, Spain) were the source of the *psy* gene from maize (GenBank: AY324431.1) and the *crtI* gene from *Pantoea ananatis* (GenBank: D90087.2, *crtI* gene location: 3582–5060 bp), respectively. For construction of the plant transformation vectors, the genes were amplified by PCR using aatB1-psy and aatB2-psy primers for *psy*, and attB1-crtI and attB2-crtI primers for *crtI.* Primers used in this study are listed in Table S4. The fragments were individually subcloned into pDONR221 via Gateway recombination^78^ with BP Clonase™ II enzyme mix and then cloned into a pK2GW7 vector (kindly given by Jörg Becker, Portugal) via GATEWAY recombination with LR Clonase™ II enzyme mix (Invitrogen). This resulted in construct pY and construct pI (Fig. 2) expressed under the Cauliflower Mosaic Virus 35 S promoter (CaMV 35 S) and kanamycin (NZYtech) as selection marker.

#### *β-carotene ketolase(crtW)*

The coding sequence for *crtW* (GenBank: AB181388.1, *crtW*) from the marine bacterium *Brevundimonas* sp. NBRC 101024 strain SD212 was codon optimized for tobacco and synthesized by GeneCust (www.genecust.com/en/) with the addition of EcoRI and BamHI restriction sites at the 5´ and 3´ ends, respectively. It included a fused sequence encoding the plastid-transit peptide from pea (*Pisum sativum*) ribulose 1,5-biphosphate carboxylase small subunit (GenBank: X00806.1). The gene was then cloned into the pTRA vector (kindly offered by Thomas Rademacher, Aachen, Germany) using the EcoRI (Thermo Scientific™) and BamHI (Thermo Scientific™) sites. The resulting construct (pW) (Fig. 2) contained the *β-carotene ketolase* gene controlled by the CaMV 35 S constitutive promoter and a 35 S terminator.

All three binary plasmids were transferred into *Agrobacterium tumefaciens* by the freeze-thaw method^79^. Constructs pY and pI were inserted into strain GV3101::pMP90 while construct pW was transferred to strain GV3101::pMP90RK.

### **Transient expression in*Nicotiana benthamiana*leaves**

Recombinant *Agrobacterium* cells, harboring either the p19 gene sequence, the empty vector pK2GW7, or one of three constructs (pY, pI, or pW) were cultured in Yeast Extract Beef (YEB) medium. The YEB medium was composed of 0.1% (w/v) of nutrient broth (BioLife Solutions), 0.1% (w/v) of yeast extract (Duchefa), 0.5% (w/v) of tryptone (NZYtech), 0.5% (w/v) of sucrose, and 2 mM of MgSO_4_, with a final pH of 7.4. The medium was supplemented with 50 mg L^−1^ of rifampicin (NZYtech) and kanamycin, and 80 mg L^−1^ of carbenicillin (NZYtech) for pW, while the other constructs were cultured with 50 mg L^−1^ of rifampicin and spectinomycin, and 25 mg L^−1^ of gentamycin. Cultures were incubated at 28 °C and 200 rpm (INNOVA) in the dark for a period of 2–3 days before being subcultured onto fresh medium. Four distinct bacterial suspensions were prepared by resuspending each transformed *Agrobacterium* (pY; pI; pW) in infiltration medium^80^ to a final optical density at 600 nm (OD_600nm_) of 0.2. For multi-combinatorial transient expression, the transformed *Agrobacteri*a were co-cultured in infiltration medium in various combinations (pY + pI; pY + pW; pI + pW; pY + pI + pW). Each one of these bacterial suspensions was co-cultured with *Agrobacterium* harboring the p19 gene, an RNA silencing suppressor (WAK97598.1), to a final OD_600nm_ of 0.1. Fully grown *N. benthamiana* leaves were punctured on the abaxial surface, and the *Agrobacterium* suspensions were introduced using a blunt-end 1 ml syringe until the entire leaf was infiltrated. Leaves were harvested 72 h post-infection and stored at −80 °C until further analysis.

### Stable transformation of tobacco BY-2 cell suspension cultures

Four-day-old tobacco BY-2 cells were transformed by co-cultivation with recombinant *A. tumefaciens*, following the protocol described by An^81^with slight modifications^82^. For multigene transformation, recombinant *A. tumefaciens* carrying individual genes were incubated together in infiltration medium for two hours before co-culturing with BY-2 cells (Table S2). After a two-day co-culture period, the cells were transferred to MS medium solidified with 0.4% gelrite (Duchefa), containing 500 mg L^−1^ ticarcillin disodium/clavulanate potassium (Timentin, Duchefa) to eliminate *Agrobacterium* and 100 mg L^−1^ kanamycin to select transformants. Cells were grown at 28 °C with a 16-hour fluorescent light photoperiod and micro-*calli* were transferred to fresh medium containing 100 mg L^−1^ kanamycin and 50% decrease of Timentin after two to four weeks^82^.

Following transformation, transgenic BY-2 cell cultures were grown as described above, under a 16-hour LED light photoperiod in supplemented MS medium containing 100 mg L^−1^ kanamycin (NZYTech). BY-2 *calli* were grown under identical conditions, except that the fluorescent light intensity was maintained at 50 µmol m^−2^ s^−1^ for *calli* growth. Wild-type and transgenic liquid cultures were subcultured into fresh medium weekly, while *calli* were subcultured monthly.

### Screening of positive transformants

Genomic DNA was extracted from tobacco BY-2 wild-type (WT) and transgenic cell lines with NZY Plant/Fungi gDNA Isolation kit (NZYTech) following the manufacturer’s instructions. PCR reactions were performed using Supreme NZYTaq II 2x Colourless Master Mix (NZYTech) with the following thermal cycling conditions: initial denaturation at 95 °C for 10 min followed by 34 cycles of denaturation (94 °C, 30 s), annealing (65–69 °C for 30 s), extension (72 °C, 15 s) followed by a final extension (72 °C, 10 min). The design of primers was carried out using the Primer3 Plus software (version 3.3.0), and primer sequences and annealing temperatures are outlined in Table S4.

### Carotenoid extraction and quantification

#### *Carotenoid profile analysis of BY-2 wild-type cells andNicotiana benthamiana leaves*

WT BY-2 cells collected during the elicitation experiment and *N. benthamiana* leaves used for transient expression were ground using liquid nitrogen. Pigments were extracted from the cells or leaves with ethanol (1:30, m/v) at 45 °C for 2 h, followed by six rounds of vortexing. The mixture was centrifuged, the supernatant was collected, and the extraction repeated until color exhaustion. The organic phase was filtered through a 0.22 μm nylon syringe filter^83^. HPLC analysis of the organic phase was carried out using a Waters Alliance System equipped with a diode array detector, with a detection range of 190 to 800 nm. A C18 reverse phase column (DeltaPaK, 5 μm particle size, 3.9 × 150 mm) was used for pigment separation. The mobile phase was a linear gradient of acetonitrile (ACN) and water with a flow rate of 0.5 mL min^−1^ (starting with 80% ACN for 1 min, increasing from 80 to 100% ACN over 8 min, maintained at 100% for 3 min, reduced to 80% over 2 min and maintained at 80% ACN for 6 min)^83^. The chromatograms were recorded at 445 nm and the carotenoids were identified by comparing retention time and UV absorption spectral properties to reference standards (kindly given by Hugo Pereira, Portugal) and previous analysis. Data processing was carried out using the automated integration software Empower 3 (SR2 Hotfix 3 Database Version 7.21.00.00).

#### Quantitative analysis of carotenoids in transgenic BY-2 cell lines

Carotenoid extraction was performed based on the methodology outlined by Stinco and co-workers^84^. The cells were lyophilized (Edwards K4 Lyophilizer) for 72 h and then ground using liquid nitrogen. A mixture of 10 mg of biomass and 500 µL of hexane: ethyl acetate (1:1) was incubated at 45 °C for 2 h, followed by six rounds of vortexing. The colored organic fraction was collected by centrifugation, and the extraction steps were repeated until color exhaustion. The organic phase was evaporated in a rotary evaporator, and the dry extract was resuspended in methanol to a final concentration of 5 mg mL^−1^. The samples were then filtered as previously described. The HPLC analysis of the collected supernatant was conducted following established protocols with slight modifications. The mobile phase was a linear gradient of acetonitrile and water, with a flow rate of 0.5 mL min^−1^ (starting at 70% ACN, increasing to 100% in 20 min, maintained at 100% for 5 min, then reduced back to 70% ACN over 2 min, and held at 70% ACN for 10 min). The chromatograms were recorded at 445 nm and, carotenoids were identified based on retention time and UV absorption spectral properties of reference standards^85^ for astaxanthin (Sigma-Aldrich), canthaxanthin (Sigma-Aldrich), and β-carotene (Sigma-Aldrich). Quantitative analysis was carried out by integrating peak areas from the chromatogram. One-way analysis of variance (ANOVA) followed by Tukey’s multiple comparison test was used to determine significant differences between the cell lines. Statistical analysis was conducted using GraphPad Prism software (Prism 8.0.2).

## Electronic supplementary material

Below is the link to the electronic supplementary material.

Supplementary Material 1

## Data Availability

Data is provided within the manuscript and supplementary information. Additional data is available from the corresponding author by reasonable request.
