# Supplementary material for: Metabolically engineered plant cell cultures as biofactories for the production of high-value carotenoids astaxanthin and canthaxanthin
Source: Sci Rep. 2025 Aug 6;15:28695. doi: 10.1038/s41598-025-11916-9 (PMC12328620; doi:10.1038/s41598-025-11916-9)
Supplement: Supplementary file 1 — Supplementary Material 1 [file 41598_2025_11916_MOESM1_ESM.pdf]

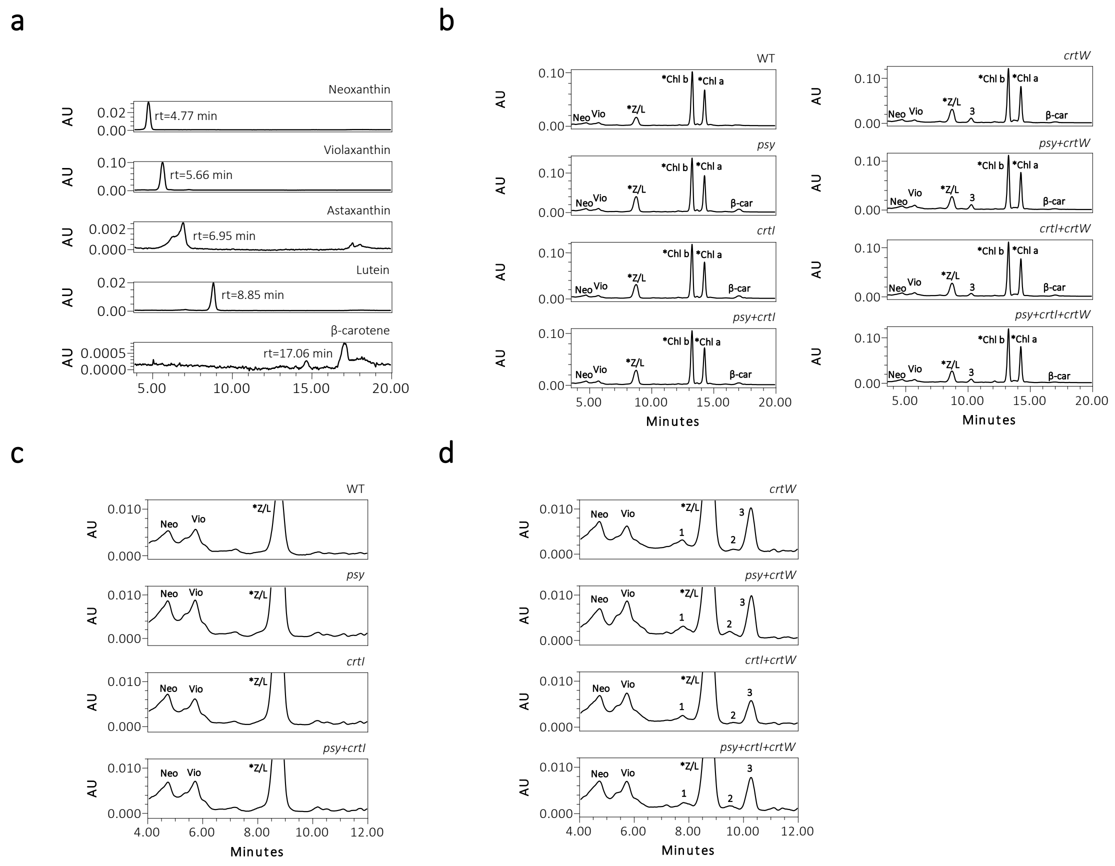

Figure S1. Carotenoid profile of *N. benthamiana* Agro-infiltrated leaves collected at 3 days post-infiltration. **(a)** Chromatograms of carotenoid standards used for peak identification including neoxanthin (Neo), violaxanthin (Vio), Astaxanthin, Lutein (L) and β-carotene (β-car). **(b)** Chromatographic profiles of infiltrated leaves, showing the identified peaks Neo, Vio and β-car, and putative lutein/zeaxanthin (Z/L*), chlorophyll a (*Chl a) and chlorophyll b (*Chl a, *Chl b). Peak identification was based on retention times and overlay with standards, as well as comparison with chromatographic profiles from Arabidopsis and reported literature. In the chromatograms close-up comparing xanthophyll- **(c)** and ketocarotenoid-producing leaves **(d)**, unidentified peaks 1, 2 and 3 are visible.

a

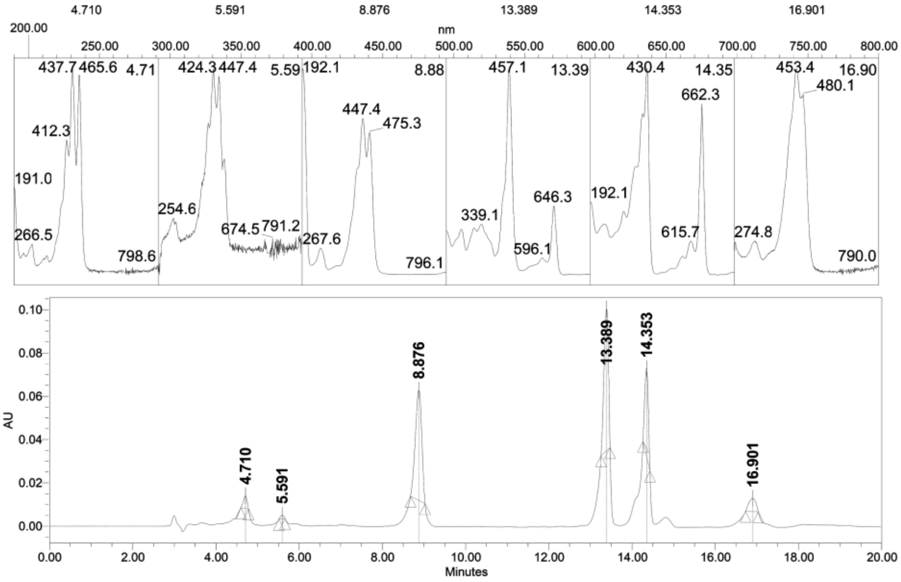

b

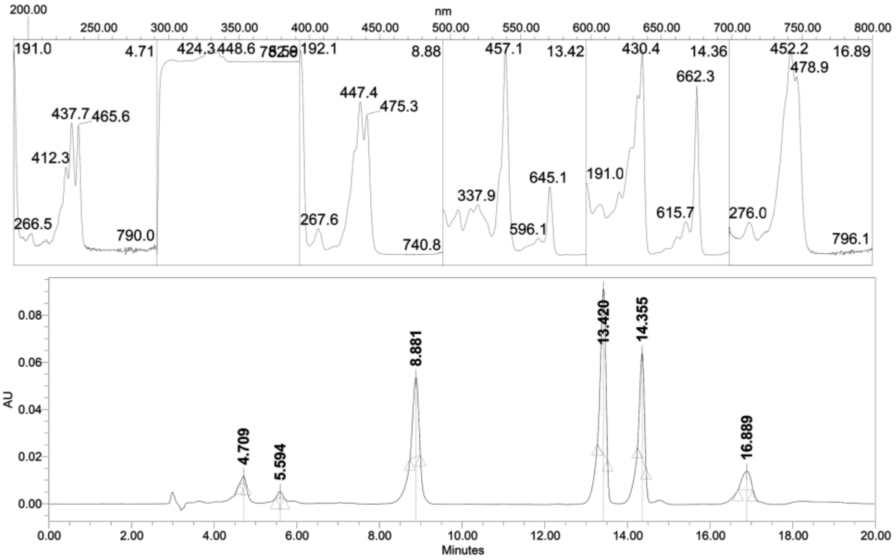

c

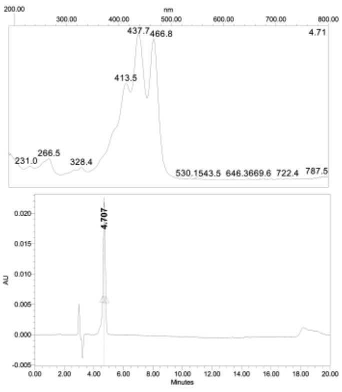

d

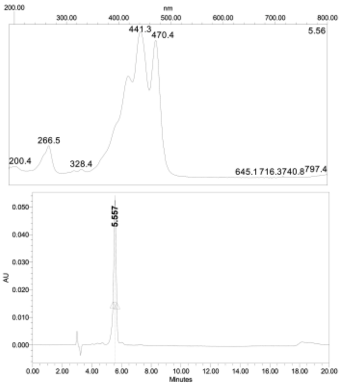

e

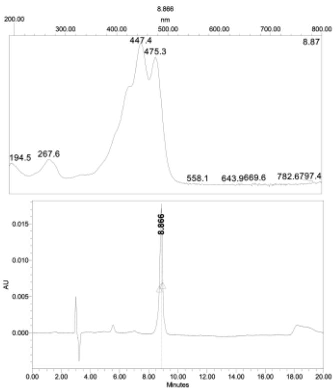

Figure S2. Pigment profile of *Arabidopsis thaliana* and *Nicotiana tabacum* leaves. HPLC absorption spectra (top) and chromatogram (bottom) of **(a)** Arabidopsis, **(b)** tobacco, and the xanthophyll standards **(c)** neoxanthin, **(d)** violaxanthin and **(e)** lutein. Retention times are indicated in the top right corner of each absorption spectrum.

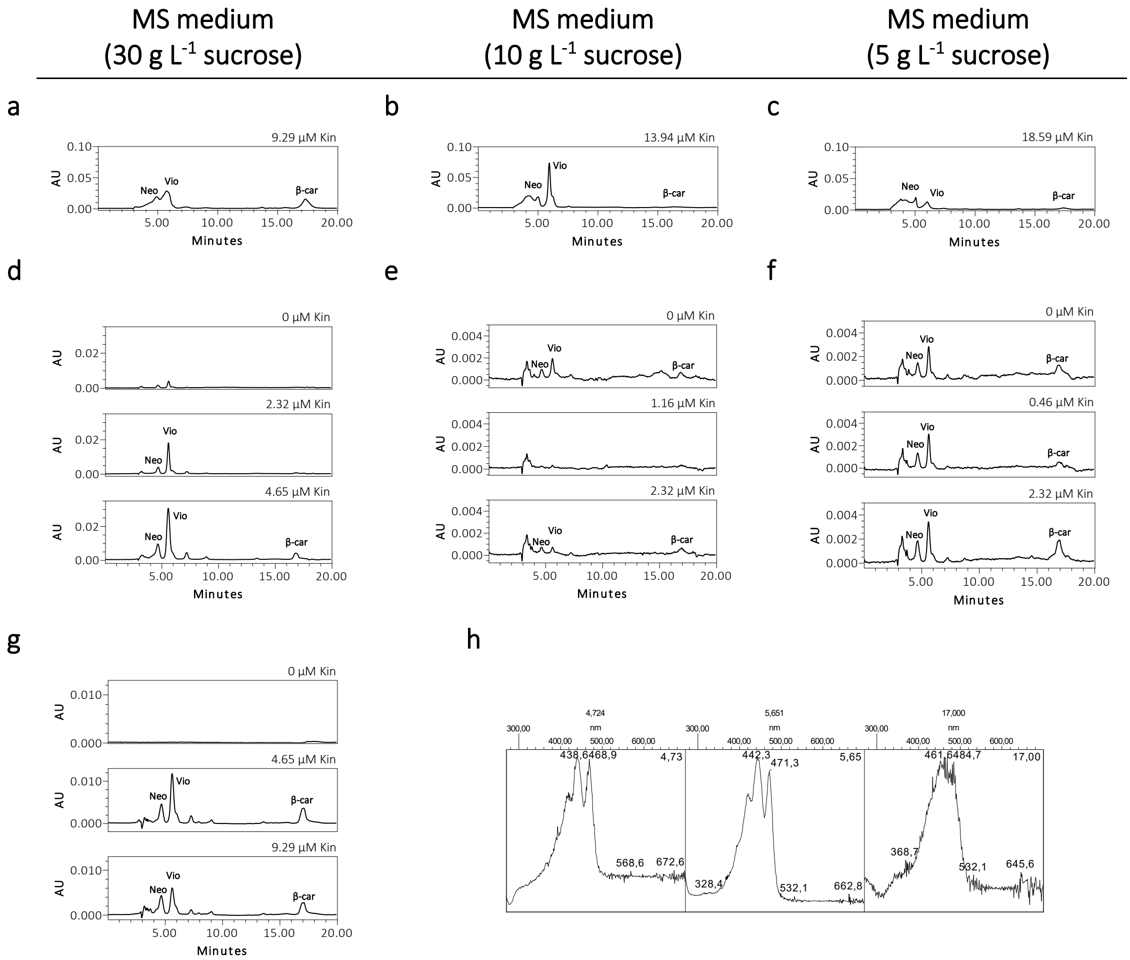

Figure S3. Carotenoid production in tobacco BY-2 WT cell suspension cultures. HPLC analysis of the carotenoid profile of cells grown under a 16 h-light photoperiod for **(a-c)** 4 months, **(d-f)** 10 months, and **(g)** 14 months. Cells were subcultured in MS medium formulated with varying amounts of sucrose concentrations (3%, 1% and 0.5%) and kinetin (Kin, 0.46 – 18.59 µM) until the 10-month time point. After this analysis, we selected cultures grown with 3% of sucrose, which showed higher yields in total carotenoid content. Sample injection volumes for HPLC were **(a-c)** 100 µL, **(d-f)** 20 µL and **(g)** 10 µL. **(h)** Representative absorption spectrum of BY-2 WT (4.65 µM Kin) ethanolic extract.

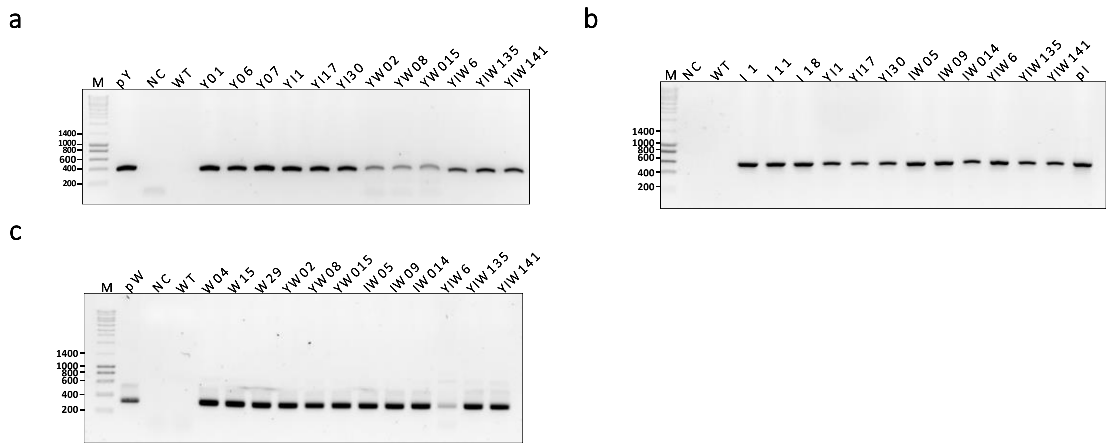

Figure S4. PCR analysis of genomic DNA of BY-2 cell lines. PCR amplified product of **(a)** maize *phytoene synthase* (*psy*) (408 bp), **(b)** bacterial *phytoene desaturase* (*crtI*) (579 bp) and **(c)** bacterial *β-carotene ketolase* (*crtW*) (331 bp) genes. M: NZYDNA Ladder III (NZYtech); NC: Negative control; pY, pI, pW: construct pY, construct pI, construct pW, respectively; WT: wild-type.

a b c

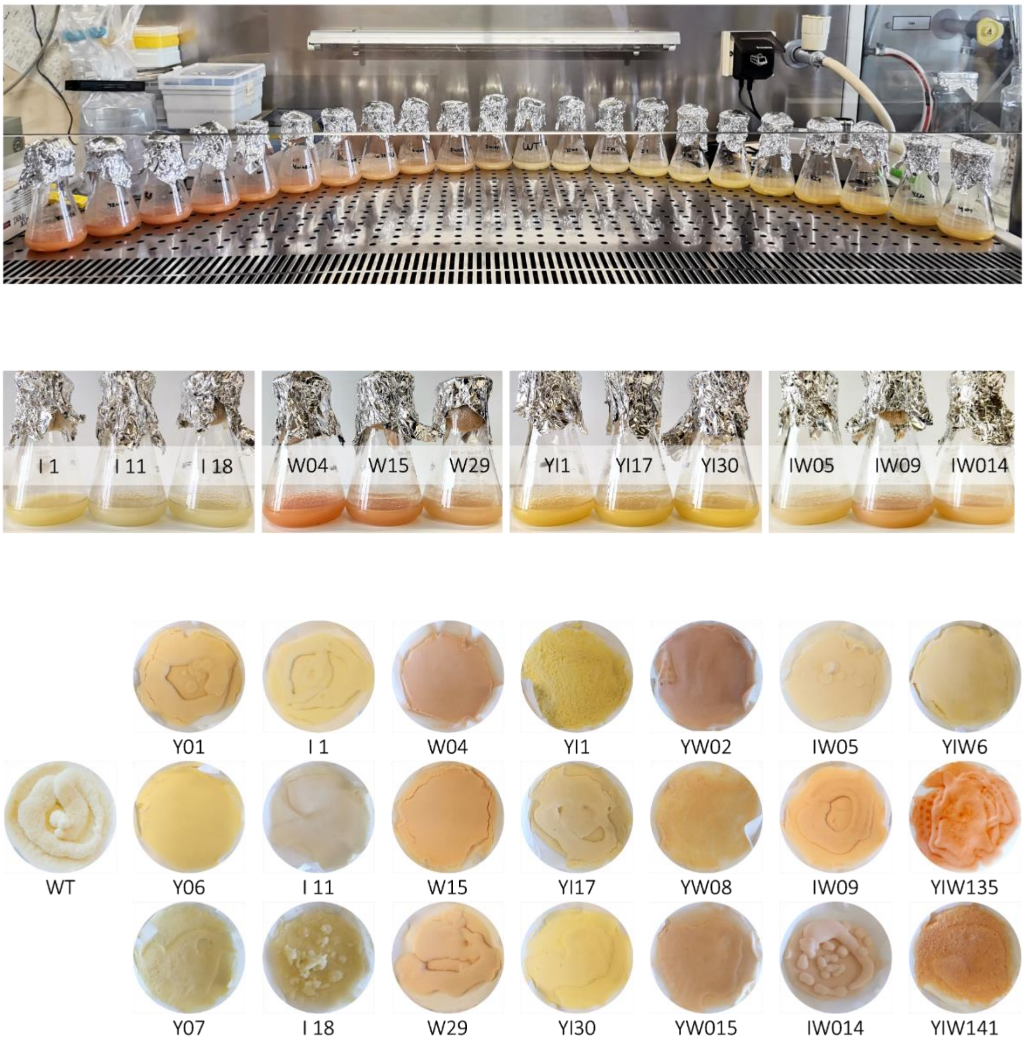

Figure S5. Tobacco BY-2 cultured cells producing ketocarotenoids and/or xanthophylls. **(a)** Cell cultures are shown according to the color observed by visual inspection, ranging from orange (left) to salmon, pale WT (center), light yellow to dark yellow (right). **(b)** Cell suspension cultures and **(c)** filtered liquid cultures used in this study.

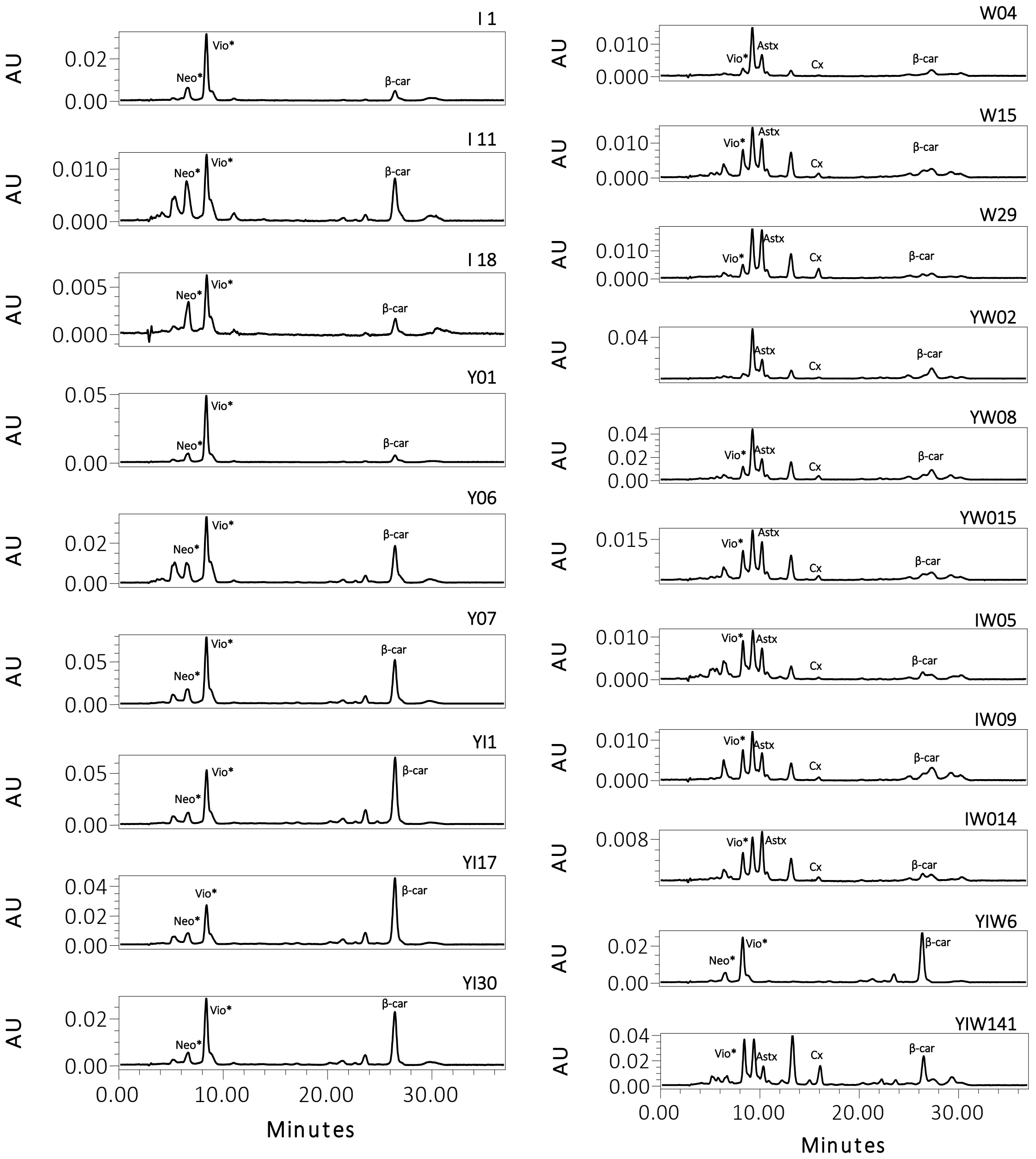

Figure S6. Carotenoid profile of tobacco BY-2 transgenic cell lines. The chromatograms were recorded at 445 nm, and the y-axis was scaled to the highest peak for clarity. Carotenoids were identified as follows: putative neoxanthin (*Neo), putative violaxanthin (*Vio), astaxanthin (Astx), canthaxanthin (Cx) and β-carotene (β-car).

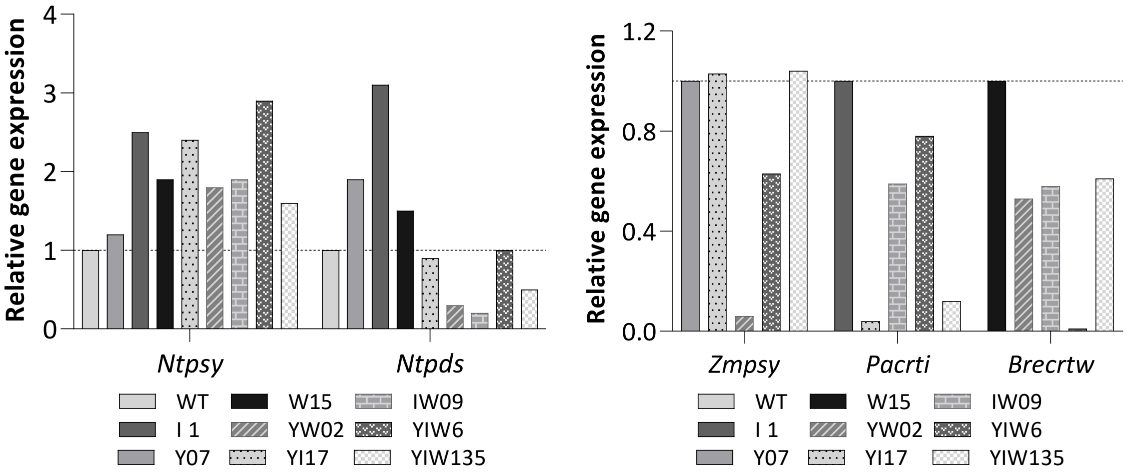

Figure S7. Semiquantitative PCR Analysis. Total RNA was extracted from tobacco BY-2 WT and selected transgenic cell lines using the Direct-zol RNA MiniPrep Kit (Zymo Research) following the manufacturer's instructions. cDNA synthesis was performed with the ImProm-II™ Reverse Transcription System (Promega). PCR reactions were carried out using Supreme NZYTaq II 2x Master Mix (NZYTech) according to the manufacturer's protocol. PCR reactions were performed using 10% (v/v) of 1:20 diluted cDNA as template, with primers at a final concentration of 0.5 µM. Cycling conditions followed the manufacturer's recommendations with the number of cycles optimized per gene: 26 cycles for *crtW*, 27 for maize *psy*, 28 for tobacco *L25* (reference gene), 29 for *crtI*, and 30 cycles for tobacco *psy* and *pds*. Primer sequences and annealing temperatures are listed in Table S5. Semi-quantitative expression levels were normalized to the endogenous reference gene *L25* to account for sample-to-sample variation. For endogenous genes, expression was reported relative to wild-type levels, while for heterologous genes, expression levels of single-transformation lines (Y07, I 1, and W15) were used as baseline controls.

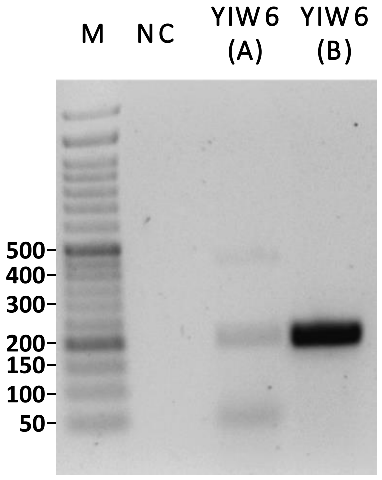

Figure S8. Detection of *crtW* transcript in YIW6 cell line by PCR. PCR was performed using 1:5 diluted cDNA (A) and undiluted cDNA (B) as templates. A total of 40 cycles was used to enhance detection sensitivity. M: NZYDNA Ladder VI (NZYtech); NC: Negative control.

Table S1. Overview of the experimental conditions employed for the carotenoid elicitation experiment in tobacco BY-2 cells.

	Sucrose (g L^{-1})	Kinetin (µM)	Inoculum (v/v)
Treatment in the dark	30	0	3%
Treatment in the light	Sucrose (g L^{-1})	Kinetin (µM)	Inoculum (v/v)
	30	0	3%
	30	4.65 – 27.88	
	10	0	3-8%
	10	1.16 – 13.94	
	5	0	3-8%
	5	2.32 – 18.59	

Table S2. Summary of the main parameters of Agrobacterium-mediated transformation of tobacco BY-2 cells.

Genotype	*A. tumefaciens* strain (binary vector)	Co-culture* OD_{600} = 0.5	Selected cell lines
Y	GV3101::pMP90 (pK2GW7:ZmPSY1 (pY))	No	Y01, Y06, Y07
I	GV3101::pMP90 (pK2GW7:PacrtI (pI))	No	I 1, I 11, I 18
W	GV3101::pMP90RK (pTRA:crtW (pW))	No	W04, W15, W29
YI	GV3101::pMP90 (pK2GW7:ZmPSY1 (pY))<br>GV3101::pMP90 (pK2GW7:PacrtI (pI))	Yes	YI1, YI17, YI30
YW	GV3101::pMP90 (pK2GW7:ZmPSY1 (pY))<br>GV3101::pMP90RK (pTRA:crtW (pW))	Yes	YW02, YW08, YW015
IW	GV3101::pMP90 (pK2GW7:PacrtI (pI))<br>GV3101::pMP90RK (pTRA:crtW (pW))	Yes	IW05, IW09, IW014
YIW	GV3101::pMP90 (pK2GW7:ZmPSY1 (pY))<br>GV3101::pMP90 (pK2GW7:PacrtI (pI))<br>GV3101::pMP90RK (pTRA:crtW (pW))	Yes	YIW6, YIW135, YIW141

* Agrobacteria cultures were individually collected by centrifugation and subsequently resuspended together in a solution of infiltration medium, which was further supplemented with 200 µM acetosyringone.

Table S3. Representative list of heterologous production of astaxanthin and canthaxanthin, highlighting the highest yields reported.

Species	Ketolase gene	Origin	Astaxanthin	Canthaxanthin	Tissue	Reported source
Arabidopsis thaliana	*bkt*[a]	*Chlamydomonas reinhardtii, Chlorella zofingiensis, Haematoccocus pluvialis*	12 – 2070 µg g^{-1} DW	200 – 600 µg g^{-1} DW	Leaf, seed	1
Brassica napus	*crbkt*[b]	*C. reinhardtii*	28.2 – 33.2 µg g^{-1} FW	4 – 4.3 µg g^{-1} FW	Cotyledon petioles	2
Daucus carota	*bkt*[b]	*H. pluvialis*	12.4 – 91.6 µg g^{-1} FW	4 – 50.1 µg g^{-1} FW	Callus, leaf, root	3
Glycine max	*bkt*[b], *crtW*[b]	*Brevundimonas* sp. SD212, *H. pluvialis*	2 – 7 µg g^{-1} DW	4 – 52 µg g^{-1} DW	Seed	4
Lotus japonicus	*crtW*	*Agrobacterium aurantiacum*	89.8 µg g^{-1} FW		Flower	5
Lactuca sativa	*crtW*[b]	*Brevundimonas* sp. SD212	178 µg g^{-1} FW	12 µg g^{-1} FW	Leaf	6
Lilium x formolongi	*crtW*[b]	*Brevundimonas* sp. SD212	0.2 – 0.5 µg g^{-1} FW	0.6 – 3.5 µg g^{-1} FW	Callus, leaf	7
Nicotiana benthamiana	*CBFD, HBFD*	*Adonis aestivalis*	0.15 – 0.99 mg g^{-1} DW	0.003 mg g^{-1} DW	Leaf	8
	crtW	*Brevundimonas* sp. SD212	0.13 – 0.4 mg g^{-1} DW	0.003 – 0.012 mg g^{-1} DW		
	crtW[b]	*Brevundimonas* sp. SD212	3.6 –14.7 µg g^{-1} DW	2.9 – 162.3 µg g^{-1} DW	Leaf	9

Species	Ketolase gene	Origin	Astaxanthin	Canthaxanthin	Tissue	Reported source
Nicotiana glauca	*crtO*[a], *crtW*	*Synechocystis, Nostoc* 73102	-	-	Flower	10
	crtW[b]	*Brevundimonas* sp. SD212	0.04 – 0.29 µg mg^{-1} DW	0.03 – 1.05 µg mg^{-1} DW	Leaf, ovary, petal	11
Nicotiana tabacum	*crtO*[a]	*H. pluvialis*	2.4 – 83.9 µg g^{-1} FW	36.4 mg g^{-1} FW	Nectary, leaf	12
	crtW[b]	*Brevundimonas* sp. SD212	1.88 – 5.44 mg g^{-1} DW	0.08 – 0.14 mg g^{-1} DW	Leaf	13
	crtW[b]	*Paracoccus sp.*	0.064 – 0.8 mg g^{-1} DW		Leaf, nectary	14
Solanum lycopersicum	*crtW*[b]	*Brevundimonas* sp. SD212	47 – 83 µg g^{-1} DW	8 – 899 µg g^{-1} DW	Fruit	15
			38.7 a 362.5 µg g^{-1} DW	19.8 – 887 µg g^{-1} DW	Fruit, leaf	16
	bkt[b]	*C. reinhardtii*	25.2 – 92.4 µg g^{-1} DW	26.1 – 84.6 µg g^{-1} DW	Fruit	17
			0.35 – 3.12 mg g^{-1} DW	1.59 – 2.3 mg g^{-1} DW	Fruit, leaf	18
	crtW[a,b]	*Brevundimonas* sp.	0.2 mg g^{-1} DW	1.5 mg g^{-1} DW	Fruit	19
Solanum tuberosum	*crtO*	*Synechocystis sp.*	0.7 – 1.8 µg g^{-1} DW	-	Tuber	20
	bkt	*H. pluvialis*	0.2 – 13.9 µg g^{-1} DW	0.2 µg g^{-1} DW	Tuber	21
	crtW[b]	*Brevundimonas* sp. SD212	0.3 – 32.3 µg mg^{-1} DW	0.06 – 0.09 µg mg^{-1} DW	Leaf, tuber	22

Species	Ketolase gene	Origin	Astaxanthin	Canthaxanthin	Tissue	Reported source
Oryza sativa	*bkt*[b]	*C. reinhardtii*	0.2 – 16.2 µg g^{-1} DW	0.04 – 25.8 µg g^{-1} DW	Grain	23
Zea mays	*bkt*[b]	*C. reinhardtii*	10.8 – 16.8 µg g^{-1} DW		Endosperm	24
Chlamydomonas reinhardtii	*bkt*[b]	*cis*	0.8 – 23.5 mg L^{-1}	0.8 – 5.2 mg L^{-1}		25
Cyanidioschyzon merolae	*bkt*[b]	*C. reinhardtii*	0.45 – 0.69 wt %	0.39 – 0.60 wt %,		26
Saccharomyces cerevisiae	*bkt, crtW*	*Brevundimonas vesicularis, Bradyrhizobium* sp., *H. pluvialis*	20 – 70 µg L^{-1}	69 – 1068 µg L^{-1}		27
	bkt[b]	*H. pluvialis*	446.4 mg L^{-1}	1 – 5 mg g^{-1} DW		28
			0.5 – 4.7 mg g^{-1} DW	-		29
Yarrowia lipolytica	*cbfd, hbfd*[b]	*A. aestivalis*	0.4 – 3.5 mg L^{-1}	-		30
	crtW[b]	*Paracoccus* sp.,	10 – 858 mg L^{-1}	-		31
	crtW	*C. reinhardtii, H. pluvialis*	0.15 – 41.3 mg g^{-1} DW	1 – 4 mg g^{-1} DW		32
	cbfd, hbfd[b]	*A. aestivalis*	trace amounts			
Corynebacterium glutamicum	*crtW*[a,b]	*Fulvimarina pelagi*	25 – 103 mg L^{-1}	10 – 75 mg L^{-1}		33

Species	Ketolase gene	Origin	Astaxanthin	Canthaxanthin	Tissue	Reported source
Escherichia coli	*crtW*[b]	*Brevundimonas* sp. SD212	10 – 1180 mg L^{-1}	170 – 1100 mg L^{-1}		34
	crtW[b]	*Brevundimonas* sp. SD212	75.6 – 610.4 µg g^{-1} DW	44.7 – 92 µg g^{-1} DW		9
	crtW[a,b]	*Anabaena variabilis*<br>*Brevundimonas* sp. SD212	70 – 320 mg L^{-1}	1 – 10.1mg L^{-1}		35
Mucor circinelloides	*bkt*[b]	*H. pluvialis*	2 – 41 µg g^{-1} DW	63 – 576 µg g^{-1} DW		36
Human embryonic kidney cells (HEK293T)	*crtW*[b], *bkt*[b]	*Brevundimonas* sp.<br>*C. reinhardtii*<br>*Haematococcus lacustris*	3.1 – 232.8 µg g^{-1} DW	29.3 – 153.1 µg g^{-1} DW		37

[a] A strain or cultivar that has been previously modified; [b] Other genes were employed in a multi-transformation approach; DW: dry weight, FW: fresh weight.

Table S4. Primers used in the present study for vector construction and amplification of *phytoene synthase*, *phytoene desaturase* and *β-carotene ketolase* gene fragments.

Primer name	Nucleotide sequence	G+C content (%)	T_{ann} (°C) / Elongation time (s)
attB1-psy	5'-GGG GAC AAG TTT GTA CAA AAA AGC AGG CTT CAT GGC CAT CAT ACT CGT AC-3'	46	60 / 90
attB2-psy	5'-GGG GAC CAC TTT GTA CAA GAA AGC TGG GTC CTA GGT CTG GCC ATT TCT CA-3'	52	
attB1-crtI	5'-GGG GAC AAG TTT GTA CAA AAA AGC AGG CTG CAT GGC TTC TAT GAT ATC CT-3'	44	56 / 90
attB2-crtI	5'-GGG GAC CAC TTT GTA CAA GAA AGC TGG GTC TCA TAT CAG ATC CTC CAG CA-3'	50	
psy_F	5'-ACA TTC AGC CAT TCA GGG ACA-3'	48	65 / 15
psy_R	5'-TTA CCC CTC TCT CTG CCT CC-3'	60	
crtI_F	5'-TAA TTG GTG CAG GCT TCG GT-3'	50	69 / 30
crtI_R	5'-ATG AGG TGG CGA AGG GAT TG-5'-	55	
crtW_F	5'-GGC TGA ACC AAG AAT TGT GCC-3'	52	68 / 10
crtW_R	5'-ACC TGG AGC TGC ATG ATG AG-3'	60	

F Forward, R reverse

Table S5. Primers used for semiquantitative PCR analysis.

Primer name	Nucleotide sequence	G+C content (%)	T_{ann} (°C)	Target gene
Ntpsy_F	5'-AGA AGC AAA TCC AGA GGG CAA G-3'	50	58	*phytoene synthase* (*psy*) from BY-2
Ntpsy_R	5'-TGG CGG TAC AAC AGC AAA GAT G-3'	50		
Ntpds_F	5'-TTG GAG CTC GAG GTC TTC TTT G-3'	50	58	*phytoene desaturase* (*pds*) from BY-2
Ntpds_R	5'-AAT CTT CTG GTC ATG GCA CTG G-3'	50		
NtL25_F	5'-CCC TCA CCA CAG AGT CTG C-3'	63	58	Ribosomal protein (*L25*) from BY-2
NtL25_R	5'-TGC TTT CTT CGT CCC ATC AGG-3'	52		
Zmpsy_F	5'-AGA GGG CGT ATG TTG GTA AAG G-3'	50	65	*phytoene synthase* (*psy*) from *Z. mays*
Zmpsy_R	5'-GAC TGG TGA TTT TTG CGG ACT C-3'	50		
PacrtI_F	5'-ATC CCC GTC TTA CTG CTT GAA C-3'	50	65	*phytoene desaturase* (*crtI*) from *P. ananatis*
PacrtI_R	5'-TTC TTC AAT GGC ACT GGG ATC G-3'	50		
BrecrtW_F	5'-ATT GTG GCT GGA TGG GGA AG-3'	55	58	*β-carotene ketolase* (*crtW*) from *Brevundimonas* sp.
BrecrtW_R	5'-TAA GCC TTC CAA CAG CAG CA-3'	50		

F Forward, R reverse

References

1. Zhong, Y. J. *et al.* Functional characterization of various algal carotenoid ketolases reveals that ketolating zeaxanthin efficiently is essential for high production of astaxanthin in transgenic *Arabidopsis*. *J. Exp. Bot.* **62**, 3659–3669 (2011).
2. Wang, J. L., Tan, S. L., He, M. X., Huang, W. & Huang, J. C. Ketocarotenoids accumulation in the leaves of engineered *Brassica napus* restricts photosynthetic efficiency and plant growth. *Environ. Exp. Bot.* **186**, 104461 (2021).
3. Jayaraj, J., Devlin, R. & Punja, Z. Metabolic engineering of novel ketocarotenoid production in carrot plants. *Transgenic Res.* **17**, 489–501 (2008).
4. Pierce, E. C. *et al.* Ketocarotenoid production in soybean seeds through metabolic engineering. *PLoS One* **10**, e0138196 (2015).
5. Suzuki, S. *et al.* Flower color alteration in Lotus japonicus by modification of the carotenoid biosynthetic pathway. *Plant Cell Rep.* **26**, 951–959 (2007).
6. Harada, H. *et al.* Construction of transplastomic lettuce (*Lactuca sativa*) dominantly producing astaxanthin fatty acid esters and detailed chemical analysis of generated carotenoids. *Transgenic Res.* **23**, 303–315 (2014).
7. Azadi, P. *et al.* Metabolic engineering of *Lilium* × *formolongi* using multiple genes of the carotenoid biosynthesis pathway. *Plant Biotechnol. Rep.* **4**, 269–280 (2010).
8. Allen, Q. M., Febres, V. J., Rathinasabapathi, B. & Chaparro, J. X. Engineering a plant-derived astaxanthin synthetic pathway into *Nicotiana benthamiana*. *Front. Plant Sci.* **12**, 1–12 (2022).
9. Nogueira, M. *et al.* Construction of a fusion enzyme for astaxanthin formation and its characterisation in microbial and plant hosts: A new tool for engineering ketocarotenoids. *Metab. Eng.* **52**, 243–252 (2019).
10. Gerjets, T., Sandmann, M., Zhu, C. & Sandmann, G. Metabolic engineering of ketocarotenoid biosynthesis in leaves and flowers of tobacco species. *Biotechnol. J.* **2**, 1263–1269 (2007).
11. Mortimer, C. L. *et al.* The formation and sequestration of nonendogenous ketocarotenoids in transgenic *Nicotiana glauca*. *Plant Physiol.* **173**, 1617–1635 (2017).
12. Mann, V., Harker, M., Pecker, I. & Hirschberg, J. Metabolic engineering of astaxanthin production in tobacco flowers. *Nat. Biotechnol.* **18**, 888–892 (2000).
13. Hasunuma, T. *et al.* Biosynthesis of astaxanthin in tobacco leaves by transplastomic engineering. *Plant J.* **55**, 857–868 (2008).
14. Ralley, L. *et al.* Metabolic engineering of ketocarotenoid formation in higher plants. *Plant J.* **39**, 477–486 (2004).
15. Nogueira, M. *et al.* Engineering of tomato for the sustainable production of ketocarotenoids and its evaluation in aquaculture feed. *Proc. Natl. Acad. Sci. U. S. A.* **114**, 10876–10881 (2017).
16. Enfissi, E. M. A. *et al.* The road to astaxanthin production in tomato fruit reveals plastid and metabolic adaptation resulting in an unintended high lycopene genotype with delayed over-ripening properties. *Plant Biotechnol. J.* **17**, 1501–1513 (2019).
17. Lin, Y., He, M., Wang, J. & Huang, J. Fruit-specific expression of *crtB*, *HpBHY*, *CrBKT* and *SlLCYB* in a special tomato landrace triggers hyper production of carotenoids in the fruit.

J. Plant Biol. **64**, 447–459 (2021).

18. Huang, J.-C., Zhong, Y.-J., Liu, J., Sandmann, G. & Chen, F. Metabolic engineering of tomato for high-yield production of astaxanthin. *Metab. Eng.* **17**, 59–67 (2013).
19. Nogueira, M. *et al.* Ketocarotenoid production in tomato triggers metabolic reprogramming and cellular adaptation: The quest for homeostasis. *Plant Biotechnol. J.* **22**, 427–444 (2024).
20. Gerjets, T. & Sandmann, G. Ketocarotenoid formation in transgenic potato. *J. Exp. Bot.* **57**, 3639–3645 (2006).
21. Morris, W. L., Ducreux, L. J. M., Fraser, P. D., Millam, S. & Taylor, M. A. Engineering ketocarotenoid biosynthesis in potato tubers. *Metab. Eng.* **8**, 253–263 (2006).
22. Mortimer, C. L. *et al.* Product stability and sequestration mechanisms in *Solanum tuberosum* engineered to biosynthesize high value ketocarotenoids. *Plant Biotechnol. J.* **14**, 140–152 (2016).
23. Zhu, Q. *et al.* From Golden Rice to aSTARice: bioengineering astaxanthin biosynthesis in rice endosperm. *Mol. Plant* **11**, 1440–1448 (2018).
24. Farré, G. *et al.* Metabolic engineering of astaxanthin biosynthesis in maize endosperm and characterization of a prototype high oil hybrid. *Transgenic Res.* **25**, 477–489 (2016).
25. Amendola, S. *et al.* Metabolic engineering for efficient ketocarotenoid accumulation in the green microalga *Chlamydomonas reinhardtii*. *ACS Synth. Biol.* **12**, 820–831 (2023).
26. Seger, M. *et al.* Engineered ketocarotenoid biosynthesis in the polyextremophilic red microalga *Cyanidioschyzon merolae* 10D. *Metab. Eng. Commun.* **17**, e00226 (2023).
27. Promdonkoy, P. *et al.* Metabolic engineering of *Saccharomyces cerevisiae* for production of canthaxanthin, zeaxanthin, and astaxanthin. *J. Fungi* **10**, 433 (2024).
28. Li, M., Zhou, P., Chen, M., Yu, H. & Ye, L. Spatiotemporal regulation of astaxanthin synthesis in *S. cerevisiae*. *ACS Synth. Biol.* **11**, 2636–2649 (2022).
29. Zhou, P., Ye, L., Xie, W., Lv, X. & Yu, H. Highly efficient biosynthesis of astaxanthin in *Saccharomyces cerevisiae* by integration and tuning of algal crtZ and bkt. *Appl. Microbiol. Biotechnol.* **99**, 8419–8428 (2015).
30. Chen, J. *et al.* Heterologous expression of the plant-derived astaxanthin biosynthesis pathway in *Yarrowia lipolytica* for glycosylated astaxanthin production. *J. Agric. Food Chem.* **71**, 2943–2951 (2023).
31. Ma, Y., Li, J., Huang, S. & Stephanopoulos, G. Targeting pathway expression to subcellular organelles improves astaxanthin synthesis in *Yarrowia lipolytica*. *Metab. Eng.* **68**, 152–161 (2021).
32. Zhu, H. Z. *et al.* Production of High Levels of 3S,3′S-Astaxanthin in *Yarrowia lipolytica* via Iterative Metabolic Engineering. *J. Agric. Food Chem.* **70**, 2673–2683 (2022).
33. Göttl, V. L. *et al.* Enhancing astaxanthin biosynthesis and pathway expansion towards glycosylated C40 carotenoids by *Corynebacterium glutamicum*. *Sci. Rep.* **14**, 1–13 (2024).
34. Gong, Z. *et al.* Coordinated expression of astaxanthin biosynthesis genes for improved astaxanthin production in *Escherichia coli*. *J. Agric. Food Chem.* **68**, 14917–14927 (2020).
35. Zhang, C., Seow, V. Y., Chen, X. & Too, H.-P. Multidimensional heuristic process for high-yield production of astaxanthin and fragrance molecules in *Escherichia coli*. *Nat. Commun.* **9**, 1858 (2018).

36. Naz, T. *et al.* Genetic modification of *Mucor circinelloides* for canthaxanthin production by heterologous expression of β-carotene ketolase Gene. *Front. Nutr.* **8**, 1–11 (2021).

37. Mohammed, Y. *et al.* Production of astaxanthin by animal cells via introduction of an entire astaxanthin biosynthetic pathway. *Bioengineering* **10**, 1–18 (2023).
